# MFG-E8 stabilized by deubiquitinase USP14 suppresses cigarette smoke-induced ferroptosis in bronchial epithelial cells

**DOI:** 10.1038/s41419-022-05455-8

**Published:** 2023-01-03

**Authors:** Yanan Cui, Lijuan Luo, Zihang Zeng, Xiangming Liu, Tiao Li, Xue He, Yiming Ma, Weiwei Meng, Huihui Zeng, Yingjiao Long, Dandan Zong, Yan Chen

**Affiliations:** 1 Department of Pulmonary and Critical Care Medicine, The Second Xiangya Hospital of Central South University, Changsha, Hunan China; 2 Research Unit of Respiratory Disease, Central South University, Changsha, Hunan China; 3 Diagnosis and Treatment Center of Respiratory Disease, Changsha, Hunan China

**Keywords:** Cell death, Respiratory tract diseases, Ubiquitylation

## Abstract

Milk fat globule epidermal growth factor 8 (MFG-E8) participates in a range of cellular processes, including reducing apoptosis and oxidative stress. However, its protective activity against cigarette smoke-induced ferroptosis in the pathogenesis of the chronic obstructive pulmonary disease (COPD) and the modulation of MFG-E8 remain unclear. Here, we showed that cigarette smoke diminished MFG-E8 protein levels but had no significant effect on its mRNA levels in lung tissues of humans and mice and in two human bronchial epithelial cell lines. MFG-E8 could attenuate ferroptosis induced by cigarette smoke extract (CSE) in vivo and in vitro. We identified ubiquitin-specific protease 14 (USP14) as a deubiquitinase of MFG-E8 in human bronchial epithelial cells. USP14 interacted with, deubiquitinated and stabilized MFG-E8. Furthermore, USP14 inhibited CSE-induced MFG-E8 proteasomal degradation. USP14 expression downregulated by CSE decreased MFG-E8 abundance and further reduced the antiferroptotic effect of MFG-E8. These findings suggest that USP14 is an essential regulator of MFG-E8 through the proteasomal pathway and that the USP14/MFG-E8 axis plays a critical role in regulating CSE-induced ferroptosis of bronchial epithelial cells.

## Introduction

Chronic obstructive pulmonary disease (COPD) is a major cause of morbidity and mortality throughout the world that induces a considerable economic and social burden [1]. Smoking is the prominent risk factor for COPD development and progression [1]. The airway epithelium is the first line of defense against pathogens and harmful particles inhaled into the lungs. Airway epithelial cell dysfunction and programmed cell death induced by cigarette smoke take part in the pathogenesis of COPD [2, 3].

Ferroptosis, first proposed by Dixon et al. in 2012, is a novel type of programmed cell death and differs from apoptosis, autophagy, and necrosis morphologically, biochemically, and genetically [4]. Ferroptosis is characterized by iron-dependent excessive accumulation of lipid peroxide and reactive oxygen species (ROS), which leads to oxidative damage of cell membranes and mitochondria [5]. Glutathione peroxidase 4 (GPx4) is the most critical ferroptosis defense gene that protects phosphatidylcholine-containing liposomes and biological membranes from peroxidative degradation [5]. Another key inhibitor of ferroptosis is solute carrier family 7 member 11 (SLC7A11), a main component of cystine/glutamate antiporter that imports cystine into cells, promotes glutathione (GSH) biosynthesis, and protects cells from oxidative stress [6]. Several studies have reported that cigarette smoke exposure could activate epithelial cell ferroptosis, participating in COPD pathogenesis [7, 8].

Milk fat globule epidermal growth factor 8 (MFG-E8) is a glycoprotein that participates in various biological and physiological processes, including enhancing the phagocytosis of apoptotic cells, reducing inflammation, promoting angiogenesis, repairing intestinal epithelium, and activating the proliferation of vascular smooth muscle cells [9–12]. It has been reported that COPD patients had a significantly lower plasma MFG-E8 level compared with controls and MFG-E8 was negatively associated with the disease severity [13]. Wang et al. further found that cigarette smoke diminished uptake of apoptotic cells by macrophages through down-regulating MFG-E8 expression [14]. However, the role of MFG-E8 in ferroptosis regulation remains unclear, and few information is focused on the modulating mechanisms of MFG-E8 protein level.

The ubiquitin–proteasome system plays a crucial role in the degradation of most cellular proteins and provides both selectivity and specificity [15]. An enzymatic cascade that involves an E1-activating enzyme, E2-conjugating enzyme, and E3-ligating enzyme connects ubiquitin (Ub) chains to target protein substrates, which are then directed to the 26 S proteasome complex where the substrates degrade into oligopeptides [16]. The protein ubiquitination can be reversed by deubiquitinases (DUBs) [17]. There are approximately one-hundred DUBs in the human proteome consisting of six families: USPs, UCHs, MJDs, OTUs, JAMMs, and MCPIP [18]. The USPs (ubiquitin-specific proteases) constitute the largest DUB family [18]. Here, we found that MFG-E8 protein might be regulated by post-translational modification and then performed a DUB screen for the regulator of MFG-E8. We identified USP14 as a potent DUB for MFG-E8 deubiquitination and proposed that cigarette smoke promoted bronchial epithelial cell ferroptosis by the inhibition of USP14/MFG-E8 axis. These results reveal an important missing piece in the dynamic regulation of MFG-E8 stability.

## Materials and methods

### Patients and samples

Lung tissue samples from COPD patients and controls who received thoracic surgery were collected from the Department of Thoracic Surgery, Second Xiangya Hospital of Central South University. COPD had been previously diagnosed according to the Global Initiative for Chronic Obstructive Lung Disease (including evaluation of symptoms, risk factors, and persistent airflow limitation defined as a post-bronchodilator forced expiratory volume in 1 s/forced vital capacity <0.70) [1]. This study was approved by the Medical Ethics Committee of the Second Xiangya Hospital of Central South University (Approval No. 2020249) and conducted in accordance with the Declaration of Helsinki. Informed consent was obtained from all involved participants.

### Preparation of cigarette smoke extract (CSE)

CSE was prepared as previously described [19] with a slight modification. Briefly, five cigarettes (Furong, Changde Cigarette Company, China; 12 mg tar, 1.1 mg nicotine, and 14 mg carbon monoxide per cigarette) were used for bubbling through 10 ml phosphate buffered saline (PBS) for animal experiments. The smoke of one cigarette was bubbled through 10 ml serum-free medium for cell experiments. The solution was then filtered through an aseptic 0.22-µm filter to obtain 100% CSE. Finally, the 100% CSE solution was diluted with PBS/culture medium to achieve the required concentration for animal and cell experiments.

### Animals

C57BL/6 wild-type (WT, MFG‐E8^+/+^) mice (6–8 weeks) were purchased from Hunan Slyke Jingda Laboratory Animal Co., Ltd. MFG-E8 knockout (KO, MFG‐E8^−/−^) mice (C57BL/6 background) were generous gifts from Professor Jungang Xie, Key Laboratory of Pulmonary Diseases of Health Ministry, Tongji Hospital, Huazhong University of Science and Technology, Wuhan, China. The gene knockout procedure was conducted as previously described [20, 21]. Tail clipping and genomic DNA analysis were performed to determine the genotypes of the offspring. The sequences of primers used for MFG‐E8 gene identification were displayed in Supplementary Table S1. Male WT mice and MFG-E8 KO mice aged 6–8 weeks were utilized for subsequent experiments.

Mice were divided into four groups: the WT-Control (WT-Ctrl) group, WT-CSE group, KO-Control (KO-Ctrl) group, and KO-CSE group, with six mice in each group. A free online randomization tool (https://www.graphpad.com/quickcalcs/randomize1.cfm) was used to assign mice to treatment groups. The murine emphysema model was established as described by He et al. [22]. The WT-CSE group and KO-CSE group were injected intraperitoneally with 0.3 ml/20 g 100% CSE at days 1, 12, and 23, while the WT-Ctrl group and KO-Ctrl group were injected with 0.3 ml/20 g PBS at days 1, 12, and 23 intraperitoneally. All mice were sacrificed to collect lung tissues on day 29. The results were blindly evaluated by investigators. All animal experiments were conducted in accordance with the Animal Ethics Committee of the Second Xiangya Hospital of Central South University (Approval No. 2020339).

### Cell culture and CSE treatment

The authenticated human bronchial epithelial cell lines (BEAS-2B and HBE) tested for mycoplasma contamination were cultured in DMEM (HyClone, USA) supplemented with 10% fetal bovine serum (Gibco, USA), 100 U/ml penicillin and 100 μg/ml streptomycin (Thermo Fisher Scientific, USA) at 37 °C in a 5% CO_2_ culture chamber. In groups treated with CSE, cells were stimulated with 5% CSE for 24 h before collection unless otherwise stated.

### Reagents

Anti-MFG-E8 (sc-271574; 1:200 dilution for immunoblotting, immunofluorescence, and immunohistochemistry; 2 µg per 1 mg of total protein for immunoprecipitation), anti-USP14 (sc-398009, 1:200 for immunoblotting, 2 µg per 1 mg of total protein for immunoprecipitation), and anti-GPx4 (sc-166570, 1:400 for immunoblotting) antibodies were purchased from Santa Cruz Biotechnology, USA. Anti-SLC7A11 (12691, 1:1000 for immunoblotting) and anti-Ub (3936, 1:1000) were from Cell Signaling Technology, USA. Anti-Flag (F7425, 1:1000) was from Sigma-Aldrich, USA. Antibodies against β-actin (60008–1-Ig, 1:5000), β-tubulin (10068-1-AP, 1:1000), and glyceraldehyde 3-phosphate dehydrogenase (GAPDH) (60004-1-Ig, 1:1000) were from Proteintech, China. Anti-USP14 (AF301319, 1:100 for immunofluorescence) was from AiFang Biological, China. Anti-GPx4 (ab125066, Abcam, UK, 1:100), and anti-SLC7A11 (26864-1-AP, Proteintech, China, 1:50) antibodies were used for immunohistochemistry (IHC) staining. Immobilized protein A/G beads (45350) were purchased from Thermo Fisher Scientific, USA. Control IgG (sc-2025) came from Santa Cruz Biotechnology, USA. Cycloheximide (CHX) (S7418), RSL3 (S8155), and Ferrostatin-1 (Fer-1) (S7243) were from Selleck, China. MG132 (HY-13259) was purchased from MedChemExpress, USA.

### Lung tissue morphometry and IHC

Paraffin-embedded lung tissues were cut into 3.5-µm thick sections and stained with hematoxylin and eosin (HE). Emphysematous changes were quantified by the values of mean linear intercept (MLI) and destructive index (DI) [22]. Five different representative nonoverlapping fields were selected from each lung section. Sections for IHC analyses were incubated with anti-MFG-E8, anti-GPx4, or anti-SLC7A11to detect the protein expression. The average optical density (AOD) was calculated by dividing the integrated optical density value by the distribution area of the target protein. The average density of five different representative nonoverlapping fields from each tissue section was used for statistical analyses.

### Recombinant human MFG-E8 protein (rhMFG-E8) administration and stable knockdown

To evaluate the biological effect of MFG-E8, BEAS-2B, and HBE cells were treated with rhMFG-E8 (2767-MF-050, R&D systems, USA) for 2 h before CSE treatment unless otherwise specified. To determine the appropriate concentration of rhMFG-E8, BEAS-2B and HBE cells were coincubated with different concentrations of rhMFG-E8. The concentration of 500 ng/ml was enough to significantly reverse the decreased levels of ferroptosis-related proteins induced by CSE (Supplementary Fig. S1) and therefore utilized in subsequent experiments. For stable knockdown, cells were infected with the lentiviruses carrying shRNA targeting human MFG-E8 or the non-targeting control lentiviral vectors (Genechem, China) in the presence of Polybrene. Forty-eight hours later, BEAS-2B and HBE cells were cultured in medium containing puromycin for the selection of stable clones. The clones stably knocking down MFG-E8 were identified by western blotting. The shRNA sequences are presented in Supplementary Table S2.

### Plasmids and RNA interference

Flag-tagged DUB overexpression plasmids were a gift from Professor Yongguang Tao, Key Laboratory of Carcinogenesis and Cancer Invasion, Ministry of Education, Department of Pathology, Xiangya Hospital, Central South University, Changsha, China. Plasmid transfection using LipoMax^TM^ (Sudgen, China) was carried out according to the manufacturer’s instructions. BEAS-2B and HBE cells were transfected with USP14 small interfering RNA (siRNA) or non-targeting negative control siRNA (RiboBio, China) using Lipofectamine^TM^ 3000 (Invitrogen, USA) according to the manufacturer’s protocol. The siRNA sequence is shown in Supplementary Table S3.

### Immunoblotting and immunoprecipitation (IP)

Total proteins from lung tissues and cells were extracted using RIPA lysis buffer (Beyotime, China). Protein concentrations were measured by a Bicinchoninic Acid protein assay kit (Thermo Fisher Scientific, USA). Equal amounts of proteins were separated using sodium dodecyl sulfate-polyacrylamide gel electrophoresis and transferred to a polyvinylidene fluoride membrane. After being blocked for 1 h, the membrane was incubated with the indicated primary antibodies followed by incubated with the related secondary antibody. Labeled proteins were detected by the ECL plus western blotting detection system (Bio-Rad, USA). For IP assays, BEAS-2B and HBE cells were lysed in IP buffer containing protease inhibitors. Cell lysates (1 mg protein) were incubated with specific primary antibodies overnight at 4 °C, followed by incubation with 40 µl of protein A/G-agarose beads for 2 h. After washing, the precipitated complex was analyzed by immunoblotting as described above.

### MFG-E8 ubiquitination assay

BEAS-2B and HBE cells were transfected with empty vectors, USP14-overexpressed plasmids, negative control siRNA, or USP14 siRNA followed by being treated with the proteasome inhibitor MG132 for 5 h. The cell extracts were subjected to IP with anti-MFG-E8 antibody and then immunoblotted with anti-Ub or anti-MFG-E8 antibody.

### Immunofluorescence (IF)

BEAS-2B and HBE cells were fixed with 4% paraformaldehyde and permeabilized with 0.2% Triton X-100. After being incubated with the indicated USP14 and MFG-E8 antibodies, cells were immunoblotted with fluorescence-conjugated secondary antibodies. The nucleus was stained with 4’6-diamidino-2-phenylindole (DAPI). Finally, images were visualized with an inverted fluorescence microscope.

### Real-time quantitative polymerase chain reaction (RT-qPCR)

Total RNA was isolated from lung tissues or cells using the TRIzol reagent (Invitrogen, USA) and reverse transcribed into complementary DNA using a RevertAid First Strand cDNA Synthesis Kit (Thermo Fisher Scientific, USA). RT-qPCR was performed using an All-in-One qPCR Mix kit (GeneCopoeia, China) according to the manufacturer’s instructions. GAPDH was used as an internal control. The sequences of all primers used are displayed in Supplementary Table S4.

### Cell viability assay

To measure the viability of BEAS-2B and HBE cells, a Cell Counting Kit‑8 (CCK‑8) assay kit (TargetMOI, USA) was used according to the manufacturer’s protocol. Briefly, cells were seeded in 96-well plates and treated with compounds at the indicated concentrations, with five biological replicates per condition. Then each well was added with 10 µl CCK-8 testing solution and cells were incubated for 2 h in the incubator. The absorbance at 450 nm was detected by a microplate reader to measure cell viability.

### Transmission electron microscopy

Mouse lung tissues and cells were fixed with 2.5% glutaraldehyde for 6 h and 1% osmium acid for 2 h followed by rinsed with PBS three times. The fixed samples were dehydrated with a graded series of ethanol and then embedded in epoxy resin. Ultrathin sections were cut into a thickness of 50–100 nm, stained with uranyl acetate and lead citrate, and observed with a Hitachi HT7700 transmission electron microscope (Hitachi, Japan).

### Measurement of lipid peroxidation

Lipid peroxidation was detected using a C11-BODIPY 581/591 probe (C10445, Invitrogen, USA). After the treatment of cells as indicated, 1 μM C11-BODIPY 581/591 was added, and the cells were incubated for 30 min. Then, cells were washed three times with PBS to remove the probes. The fluorescence of C11-BODIPY 581/591 was analyzed by flow cytometry (A00-1-1102, Beckman, USA).

### Measurement of iron

The total iron and Fe^2+^ in each cell line were measured with the Iron Assay Kit (MAK025, Sigma-Aldrich, USA) following the manufacturer’s protocols. PERLS-Diaminobenzidine (DAB) staining was used to detect iron accumulation in paraffin-embedded lung sections. Briefly, after dewaxing, staining, and color development, the lung tissues with iron expression presented brown deposits.

### Statistical analysis

All quantified data were expressed as mean ± standard deviation (SD). Statistical comparisons were performed using the one-way ANOVA or the *t* test as appropriate. GraphPad Prism 8.0 was used to analyze the data. A two-sided *P* value <0.05 was considered statistically significant.

## Results

### Cigarette smoke diminishes MFG-E8 protein expression in vivo and in vitro

**Fig. 1:**
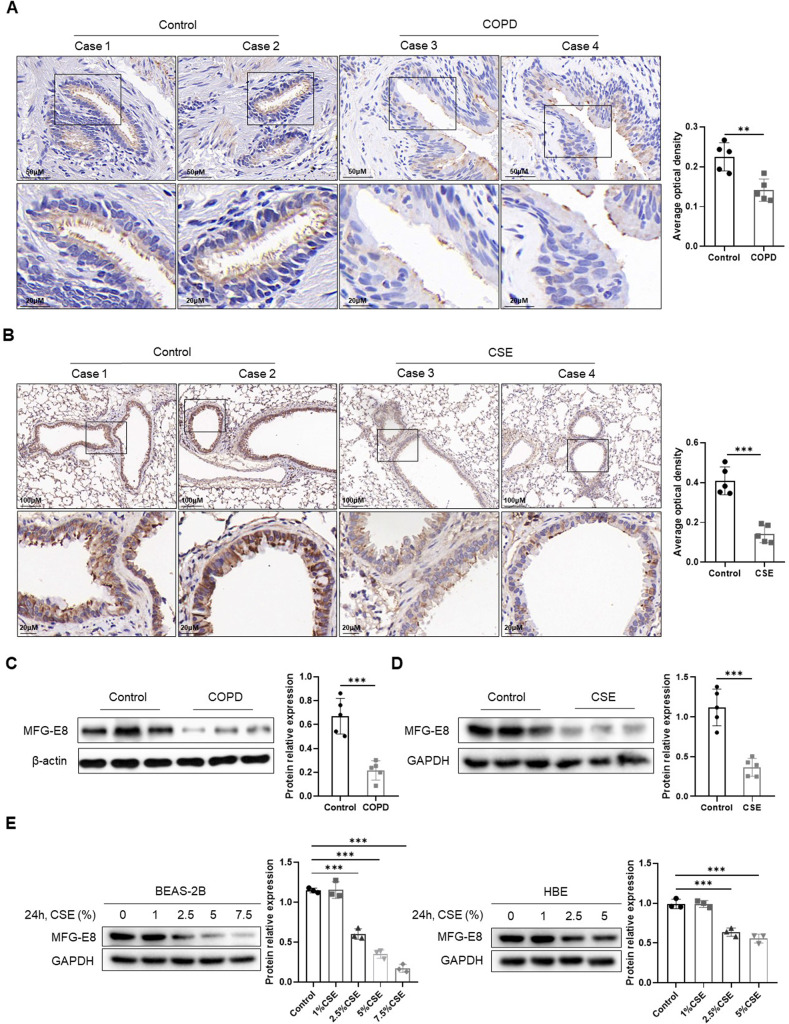
Cigarette smoke exposure diminishes MFG-E8 protein expression in vivo and in vitro. **A** IHC staining of MFG-E8 in lung tissues of COPD patients and healthy controls. The average optical density of MFG-E8 in human lung tissues is plotted in the right-hand panel. **B** IHC staining of MFG-E8 in lung tissues of mice. Control: WT mice exposed to PBS. CSE: WT mice exposed to CSE. The average optical density of MFG-E8 in mouse lung tissues is plotted in the right-hand panel. **C** Western blot analyses of MFG-E8 protein levels in lung tissues of COPD patients and healthy controls. **D** Western blot analyses of MFG-E8 protein levels in lung tissues of mice. Control: WT mice exposed to PBS. CSE: WT mice exposed to CSE. **E** Western blot analyses of MFG-E8 protein levels in BEAS-2B cells and HBE cells exposed to CSE. Data are presented as the mean ± SD. ^**^P < 0.01, compared between the marked groups. ^***^P < 0.001, compared between the marked groups.

IHC staining was performed to detect the expression and localization of MFG-E8. MFG-E8 was widely distributed in the airway epithelium, and its abundance was significantly downregulated in COPD patients compared with the controls (Fig. 1A). In mouse lung tissues, CSE exposure significantly reduced the expression of MFG-E8 in airway epithelial cells (Fig. 1B). Western blotting showed that MFG-E8 protein levels were aberrantly decreased in the lung tissue of COPD patients and mice exposed to CSE (Fig. 1C, D). Moreover, we conducted CSE treatment in human bronchial epithelial cell lines (BEAS-2B and HBE cells). The immunoblotting analysis showed that CSE decreased MFG-E8 protein levels in a concentration-dependent manner (Fig. 1E). 5% CSE treatment (24 h) was selected for subsequent experiments. These data indicated that cigarette smoke diminished MFG-E8 protein levels both in vivo and in vitro.

### MFG-E8 regulates CSE-induced ferroptosis in human bronchial epithelial cells

**Fig. 2:**
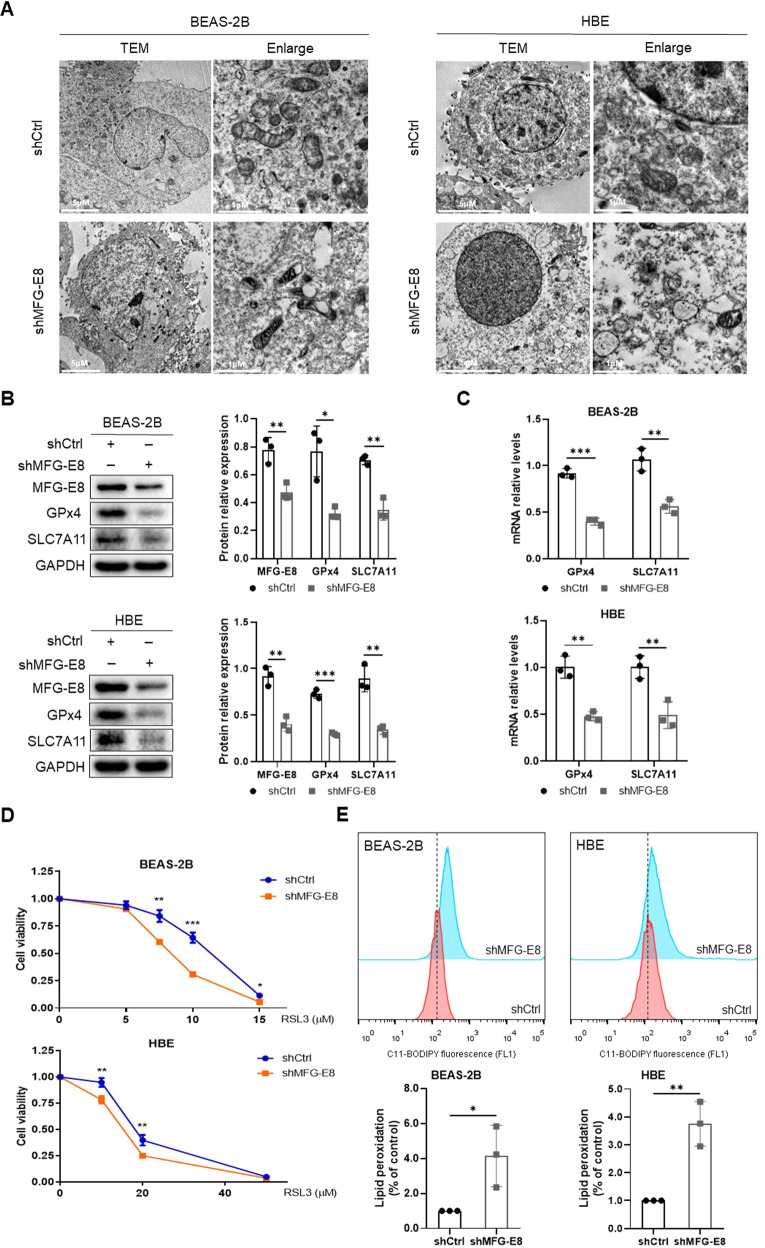
MFG-E8 silencing induces ferroptosis in BEAS-2B cells and HBE cells. **A** The morphological changes of mitochondria observed by transmission electron microscopy. **B** Ferroptosis-related proteins (GPx4 and SLC7A11) were detected by western blot. **C** Levels of GPx4 and SLC7A11 mRNA. **D** Cell viability of MFG-E8 knockdown cells incubated with different concentrations of RSL3 for 24 h using CCK-8 assays. **E** Lipid peroxidation determined by C11-BODIPY staining. Data are presented as the mean ± SD of three independent experiments. ^*^P < 0.05, compared between the marked groups. ^**^P < 0.01, compared between the marked groups. ^***^P < 0.001, compared between the marked groups.

To investigate the effect of MFG-E8 on ferroptosis in vitro, we constructed a stable MFG-E8 knockdown cell line. As shown in Fig. 2A, MFG-E8 knockdown led to mitochondrial shrinkage, membrane density increasing, and cristae rupture in BEAS-2B and HBE cells. The protein and mRNA expression levels of GPx4 and SLC7A11 were significantly decreased after MFG-E8 silencing (Fig. 2B, C). Viability curves demonstrated that MFG-E8 knockdown cells exhibited higher sensitivity to RSL3-induced ferroptosis (Fig. 2D). Furthermore, C11-BODIPY staining showed MFG‐E8 silencing apparently increased the lipid peroxidation in BEAS-2B and HBE cells (Fig. 2E).

**Fig. 3:**
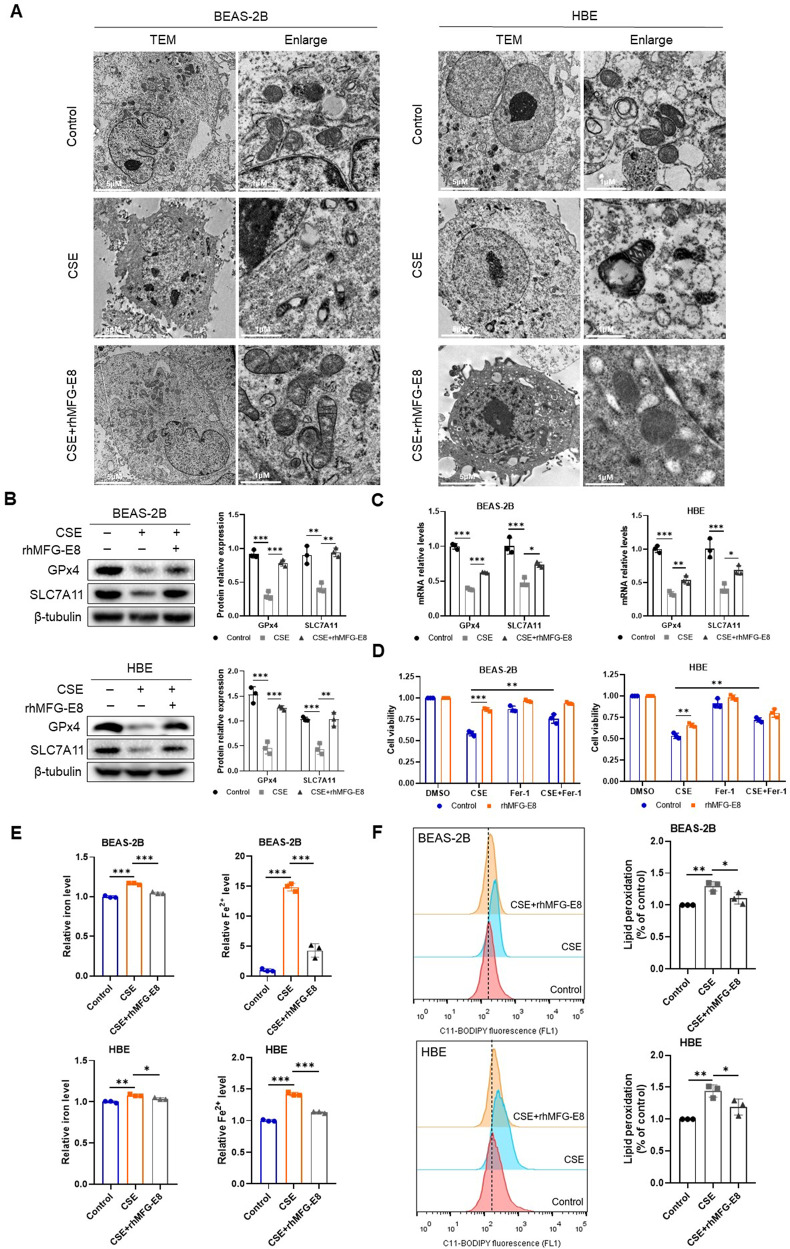
Administration of rhMFG-E8 ameliorates ferroptosis induced by CSE in BEAS-2B cells and HBE cells. **A** The morphological changes of mitochondria observed by transmission electron microscopy. **B** Ferroptosis-related proteins (GPx4 and SLC7A11) were detected by western blot. **C** Levels of GPx4 and SLC7A11 mRNA. **D** Cell viability using CCK-8 assays. Cells pretreated with rhMFG-E8 were exposed to CSE and/ or Fer-1 (5 μM for BEAS-2B cells and 10 μM for HBE cells) for 24 h. **E** Levels of total iron and ferrous iron. **F** Lipid peroxidation determined by C11-BODIPY staining. Data are presented as the mean ± SD of three independent experiments. ^*^P < 0.05, compared between the marked groups. ^**^P < 0.01, compared between the marked groups. ^***^P < 0.001, compared between the marked groups.

In order to verify whether MFG-E8 plays a role in alleviating ferroptosis of CSE-exposed bronchial epithelial cells, the cells were pretreated with rhMFG-E8 followed by CSE stimulation. Through transmission electron microscopy, deformation and vacuolization of mitochondria with an increased membrane density were observed in CSE-treated cells, and these morphological alterations were markedly ameliorated in rhMFG-E8-pretreated cells (Fig. 3A). Figure 3B, C shows CSE significantly suppressed GPx4 and SLC7A11 expressions, which were dramatically reversed by pretreatment with rhMFG-E8. We found that CSE significantly reduced cell viability, but the administration of ferroptosis inhibitor Fer-1 or rhMFG-E8 decreased CSE-induced growth inhibition (Fig. 3D). Furthermore, CSE increased the intracellular concentrations of iron, which could be reversed by pretreatment with rhMFG-E8 (Fig. 3E). Pretreatment with rhMFG-E8 notably attenuated the lipid peroxidation induced by CSE (Fig. 3F). Overall, these results suggested that MFG-E8 significantly reversed the ferroptosis induced by CSE.

### MFG-E8 regulates CSE-induced ferroptosis in mice

**Fig. 4:**
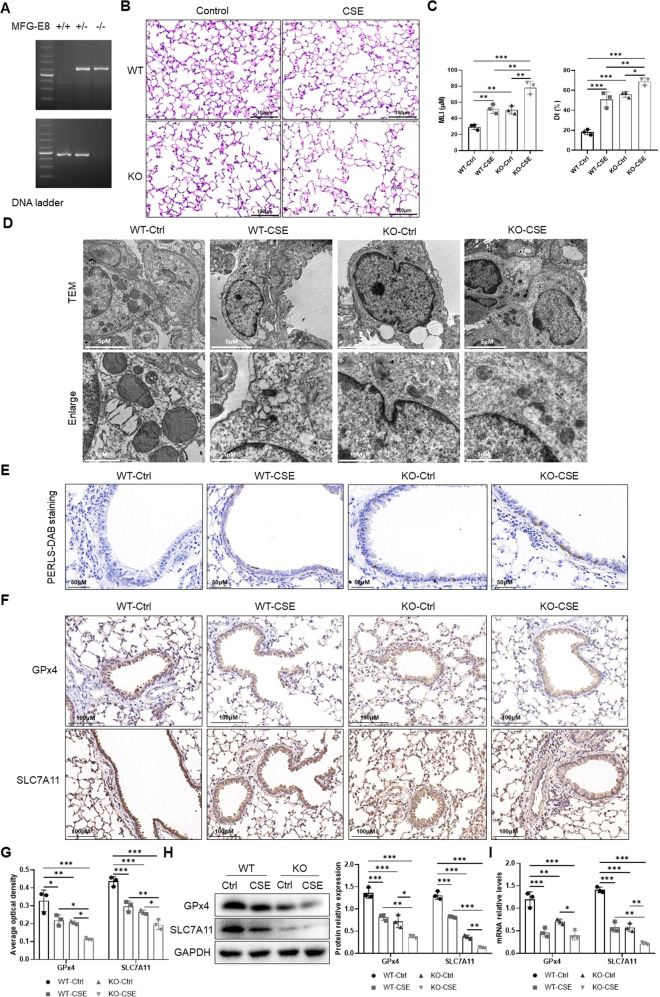
MFG‐E8 gene knockout promotes CSE-induced ferroptosis in mouse lung tissues. **A** MFG‐E8 gene identification in C57BL/6 mice. **B** HE staining of lung slides. **C** Morphometric measurements of MLI (μm) and DI (%). WT-Ctrl: WT mice exposed to PBS. WT-CSE: WT mice exposed to CSE. KO-Ctrl: MFG-E8 KO mice exposed to PBS. KO-CSE: MFG-E8 KO mice exposed to CSE. **D** The morphological changes of mitochondria observed by transmission electron microscopy. **E** Ferric iron (brown) deposits stained with PERLS-DAB staining. **F** IHC staining of ferroptosis-related proteins (GPx4 and SLC7A11). **G** The average optical densities of GPx4 and SLC7A11 in IHC staining. **H** Western blot analyses of GPx4 and SLC7A11 protein levels. **I** Levels of GPx4 and SLC7A11 mRNA. Data are presented as the mean ± SD of three independent experiments. ^*^P < 0.05, compared between the marked groups. ^**^P < 0.01, compared between the marked groups. ^***^P < 0.001, compared between the marked groups.

We identified the MFG-E8 KO mice to further assess the effect of MFG-E8 on ferroptosis in lung tissues of CSE-exposed mice (Fig. 4A). HE staining was performed to detect histological changes in mouse lung tissues (Fig. 4B). Compared with the WT-Ctrl group, the MLI and DI values were significantly increased in the WT-CSE group, suggesting successful modeling of emphysema mice (Fig. 4C). These values were dramatically increased in the KO-CSE group compared with the WT-CSE group (Fig. 4C). We used transmission electron microscopy to observe the morphological features of the mitochondria (Fig. 4D). Obvious shrinkage of mitochondria with an increased membrane density and reduction of mitochondrial cristae were observed in the WT-CSE group. MFG-E8 deficiency aggravated these morphological changes in CSE-exposed mice, accompanied by more rupture of mitochondrial cristae and membrane. PERLS-DAB staining disclosed that the strongest staining of iron deposition, along with more stained areas in lung sections, were detected in the KO-CSE group (Fig. 4E). IHC staining showed a lower expression of GPx4 and SLC7A11 in the KO-CSE group than that in the WT-CSE group (Fig. 4F, G). A similar trend could be observed in immunoblotting and RT-qPCR analyses (Fig. 4H, I). These results indicated that MFG-E8 deficiency promoted CSE-induced ferroptosis in mouse lung tissues.

### Cigarette smoke does not alter MFG-E8 mRNA levels in vivo and in vitro

**Fig. 5:**
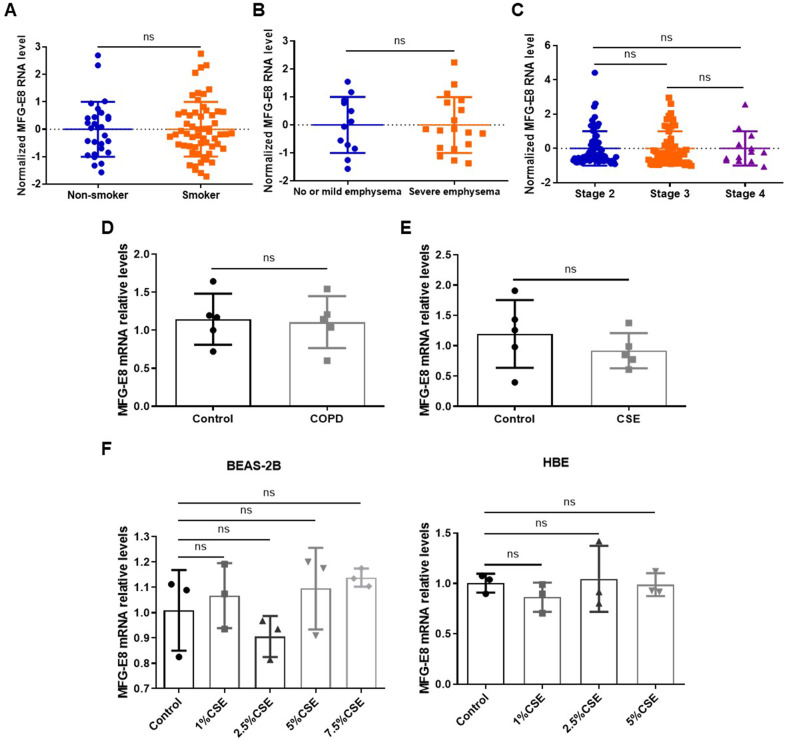
Cigarette smoke exposure shows no significant effect on MFG-E8 mRNA levels in vivo and in vitro. **A** MFG-E8 RNA levels in small airway epithelium were similar between 28 samples of non-smokers and 58 samples of smokers from GEO Profiles. **B** MFG-E8 RNA levels in human lung tissues were similar between 12 samples with no or mild emphysema and 18 samples with severe emphysema from GEO Profiles. **C** MFG-E8 RNA levels in sputum were similar between 71 stage 2 COPD samples, 59 stage 3 COPD samples, and 13 stage-4 COPD samples from GEO Profiles. **D** Levels of MFG-E8 mRNA in lung tissues of COPD patients and healthy controls. **E** Levels of MFG-E8 mRNA in lung tissues of mice. Control: WT mice exposed to PBS. CSE: WT mice exposed to CSE. **F** Levels of MFG-E8 mRNA in BEAS-2B cells and HBE cells exposed to CSE. Data are presented as the mean ± SD.

RNA expression data were downloaded from the GEO Profiles (https://www.ncbi.nlm.nih.gov/geoprofiles) on December 18, 2021. MFG-E8 expression levels in small airway epithelium were similar between non-smokers and smokers (Fig. 5A). MFG-E8 expression levels in human lung tissues were similar between subjects with no or mild emphysema and those with severe emphysema (Fig. 5B). MFG-E8 RNA levels in sputum had no association with the severity of COPD (Fig. 5C). RT-qPCR results showed that there was no significant difference in the expression of MFG-E8 mRNA between COPD patients and the controls (Fig. 5D). In addition, CSE exposure showed no significant effect on MFG-E8 mRNA level in mouse lung tissues and two bronchial epithelial cell lines (Fig. 5E, F), indicating that CSE probably regulated MFG-E8 expression at the post-transcriptional level.

### MFG-E8 is degraded by the proteasome

**Fig. 6:**
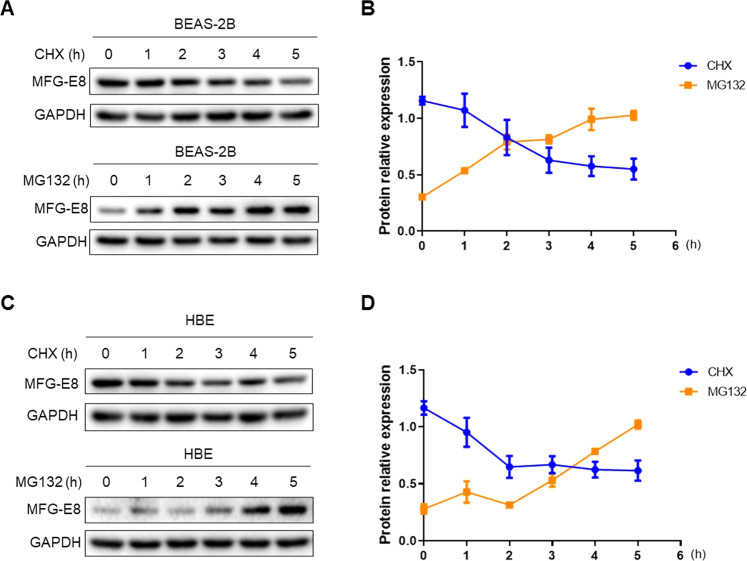
MFG-E8 is degraded by the proteasome. **A** BEAS-2B cells were treated with 10 μg/ml CHX or 5 μM MG132 for various time points prior to immunoblotting analyses of MFG-E8 protein levels. **B** The densitometric changes of MFG-E8 protein expression in BEAS-2B cells were plotted. **C** HBE cells were treated with 20 μg/ml CHX or 10 μM MG132 for various time points prior to immunoblotting analyses of MFG-E8 protein levels. **D** The densitometric changes of MFG-E8 protein expression in HBE cells were plotted. Data are presented as the mean ± SD of three independent experiments.

To investigate whether MFG-E8 is unstable and supposed to be degraded, we first detected the protein stability of MFG-E8. BEAS-2B cells were treated with CHX to inhibit protein synthesis and MFG-E8 protein levels were then analyzed by immunoblotting. The results showed that CHX diminished MFG-E8 expression in a time-dependent manner (Fig. 6A, B). We further found that MFG-E8 degradation could be rescued by the proteasome inhibitor MG132 (Fig. 6A, B). Similar phenomena were also observed in HBE cells (Fig. 6C, D), suggesting MFG-E8 was an unstable protein that could be degraded by the proteasome.

### USP14 maintains MFG-E8 stability

**Fig. 7:**
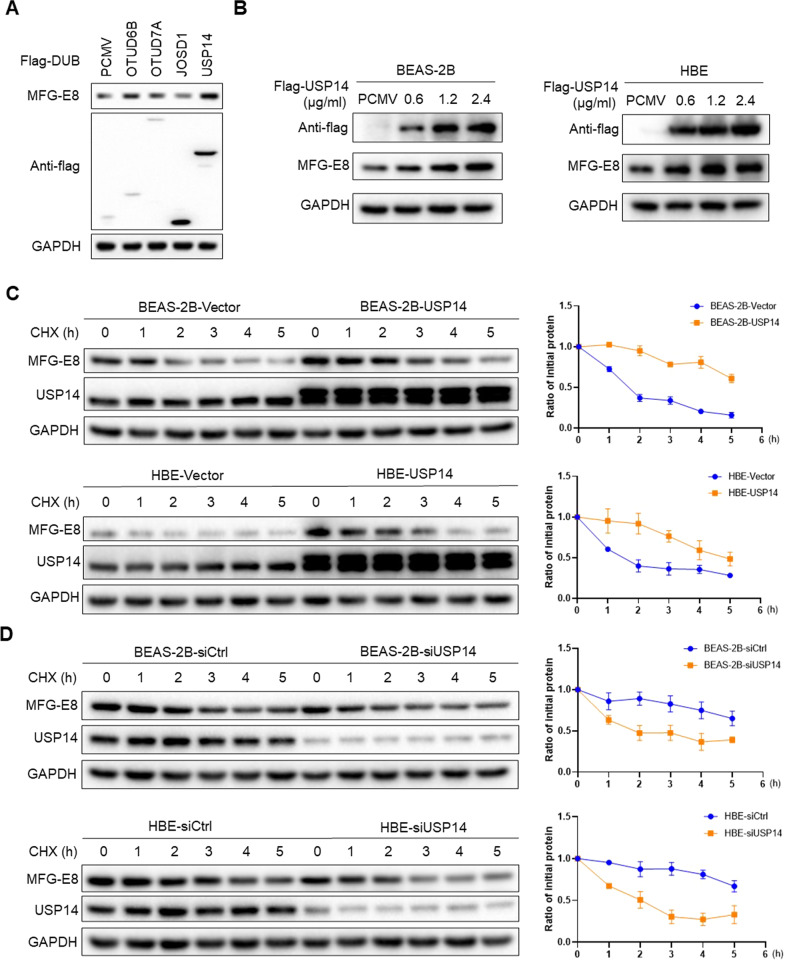
USP14 stabilizes MFG-E8 in BEAS-2B cells and HBE cells. **A** The indicated deubiquitinases were each transfected into BEAS-2B cells. Forty-eight hours later, cell lysates were subjected to western blot. **B** Increasing concentrations of USP14 plasmids were transfected into BEAS-2B cells and HBE cells. MFG-E8 protein expression was detected. **C** Empty vectors or USP14-overexpressed plasmids (2 μg/ml) were transfected into BEAS-2B cells and HBE cells. The cells were then treated with CHX for various time points prior to immunoblotting analyses. Quantification of MFG-E8 levels relative to GAPDH is shown in the right-hand panel. **D** Negative control siRNA or USP14 siRNA (50 nM) were transfected into BEAS-2B cells and HBE cells. The cells were then treated with CHX for various time points prior to immunoblotting analyses. Quantification of MFG-E8 levels relative to GAPDH was shown in the right-hand panel. Data are presented as the mean ± SD of three independent experiments.

To identify the DUB responsible for MFG-E8, several DUBs were overexpressed in BEAS-2B cells, and the expression of MFG-E8 was detected. This screen showed that USP14 remarkably upregulated the MFG-E8 level (Fig. 7A). USP14 overexpression resulted in MFG-E8 elevation in a dose-dependent manner in both BEAS-2B and HBE cells (Fig. 7B). To prove that USP14 could promote MFG-E8 stability, CHX was applied to the control cells and cells overexpressing USP14 for the indicated times and MFG-E8 stability was determined. The MFG-E8 protein level decreased more slowly in USP14-overexpressed cells than in the control cells (Fig. 7C). In addition, we added CHX to control cells and cells depleted of USP14. The MFG-E8 protein level reduced faster after silencing USP14 (Fig. 7D), indicating that the stability of MFG-E8 was decreased in cells depleted of USP14. Collectively, our data indicated that USP14 specifically stabilized MFG-E8 protein in BEAS-2B cells and HBE cells.

### USP14 interacts with and deubiquitinates MFG-E8

**Fig. 8:**
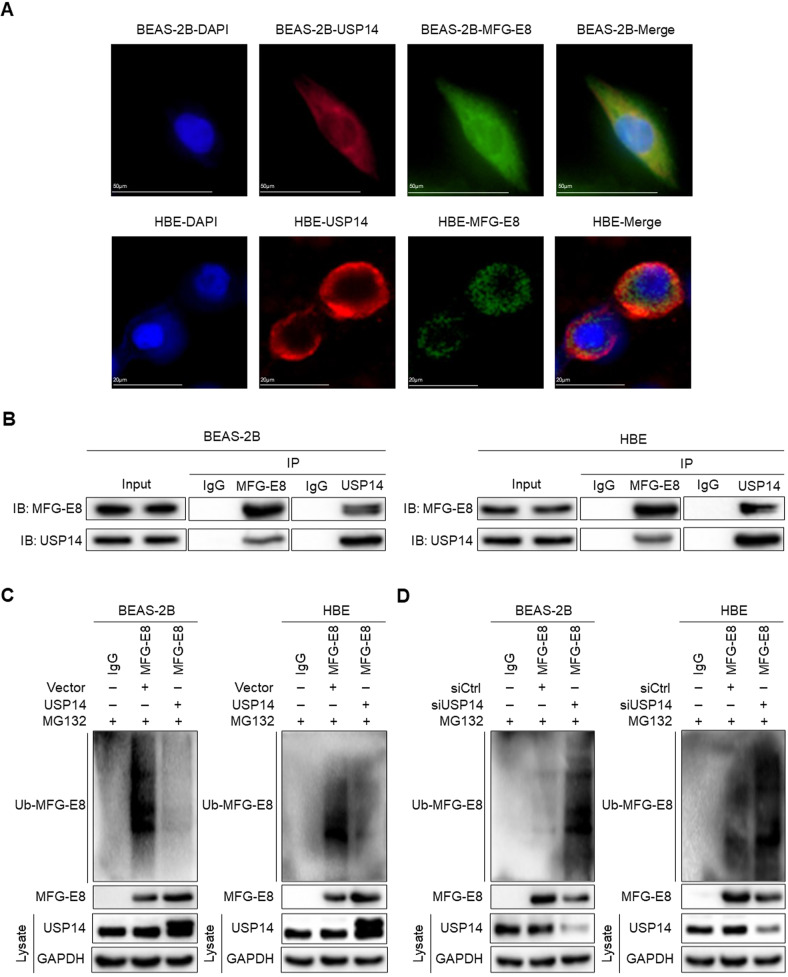
USP14 interacts with and deubiquitinates MFG-E8 in BEAS-2B cells and HBE cells. **A** Immunofluorescence staining and co-localization analyses of USP14 (red) and MFG-E8 (green). DAPI was used to dye the nucleus. **B** Cell lysates were subject to immunoprecipitation with control IgG, MFG-E8, or USP14 antibodies. The immunoprecipitates were then blotted. **C** Empty vectors or USP14-overexpressed plasmids (2 μg/ml) were transfected into cells. Equal amount of cell lysates (1 mg of total protein) were subjected to MFG-E8 immunoprecipitation followed by immunoblotting as indicated. The efficacy of USP14 overexpression in each group was determined by immunoblotting as indicated. **D** Negative control siRNA or USP14 siRNA (50 nM) were transfected into cells. Equal amount of cell lysates (1 mg of total protein) were subjected to MFG-E8 immunoprecipitation followed by immunoblotting as indicated. The efficacy of USP14 knockdown in each group was determined by immunoblotting as indicated. All panels are representative results of three independent experiments.

IF was conducted to explore the relationship between USP14 and MFG-E8. The merged figures indicated that USP14 and MFG-E8 coexisted in the cytoplasm (Fig. 8A), suggesting that USP14-mediated MFG-E8 protein upregulation might mainly occur in the cytoplasm. To further elucidate the direct binding of USP14 and MFG-E8, endogenous USP14 and MFG-E8 proteins were co-immunoprecipitated from lysates of two cell types. Results showed that MFG-E8 and USP14 were readily co-immunoprecipitated with each other. MFG-E8 was detected in the anti-USP14 immunoprecipitates, and in turn, USP14 was detected in the anti-MFG-E8 immunoprecipitates (Fig. 8B).

To directly detect whether USP14 regulates MFG-E8 via deubiquitination, endogenous polyubiquitinated MFG-E8 protein was immunoprecipitated with anti-MFG-E8 antibody and examined using anti-Ub antibody. Overexpression of USP14 substantially decreased the ubiquitination of endogenous MFG-E8 in BEAS-2B and HBE cells (Fig. 8C). Conversely, depletion of endogenous USP14 in BEAS-2B and HBE cells dramatically elevated endogenous MFG-E8 ubiquitination (Fig. 8D). Taken together, USP14 was a specific DUB for MFG-E8 and stabilized MFG-E8 protein through deubiquitination.

### USP14 expression is downregulated after CSE exposure

The combination of Western blotting and RT-qPCR was used to detect the effect of CSE on the expression of USP14 in BEAS-2B cells and HBE cells. Our data showed that CSE significantly decreased USP14 mRNA and protein levels, both in a concentration-dependent manner (Supplementary Fig. S2A, B). Further experiments showed that, in the lung tissue of mice exposed to CSE, USP14 protein levels were also aberrantly decreased (Supplementary Fig. S2C).

### USP14 inhibits CSE-induced MFG-E8 proteasomal degradation

**Fig. 9:**
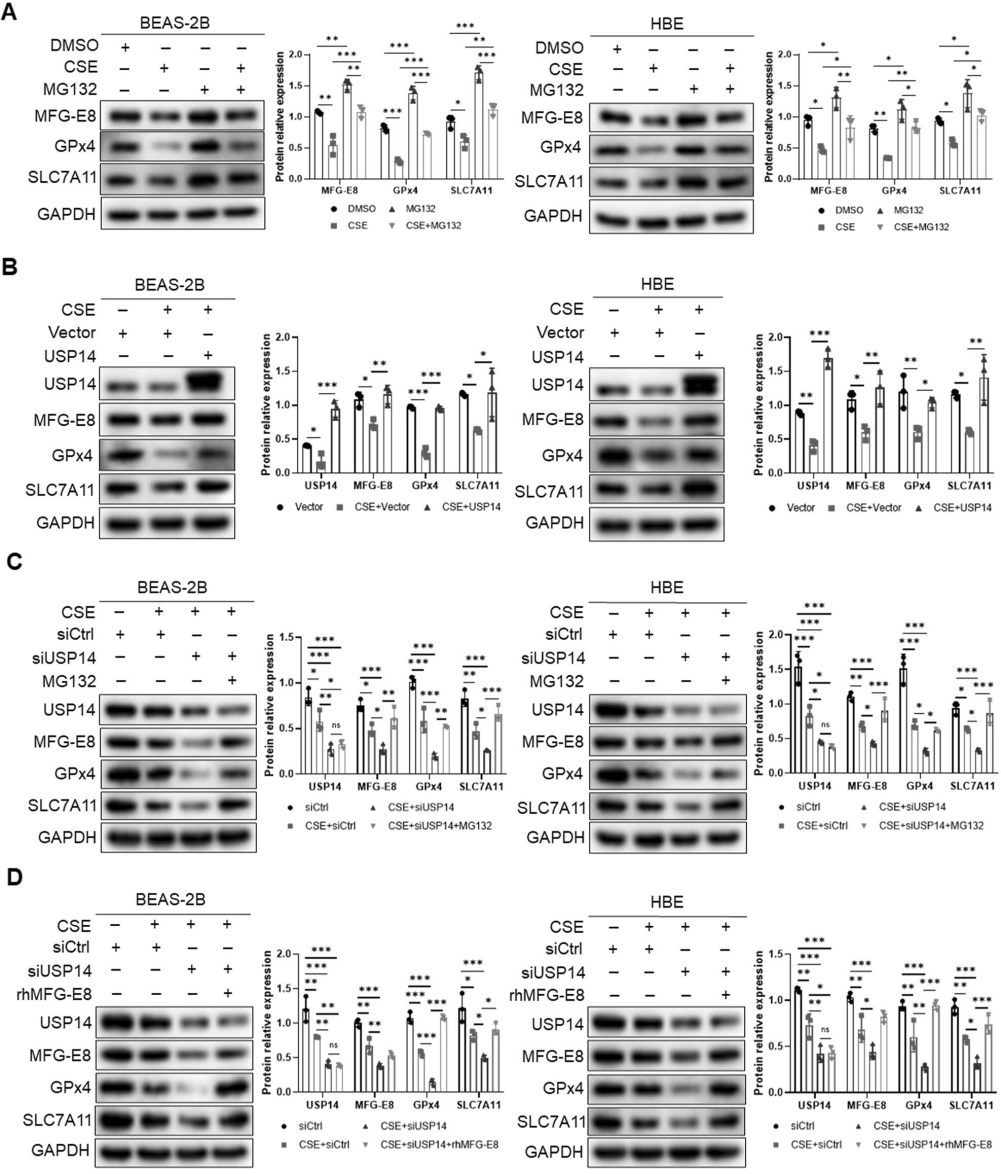
USP14/MFG-E8 axis involves in CSE-induced ferroptosis in BEAS-2B cells and HBE cells. **A** Cells were stimulated with CSE for 24 h and/ or MG132 for 5 h before collection prior to immunoblotting for MFG-E8, GPx4, and SLC7A11. **B** Empty vectors or USP14-overexpressed plasmids (2 μg/ml) were transfected into cells. Before the cells were collected, the transfected cells were stimulated with CSE for 24 h. USP14, MFG-E8, GPx4, and SLC7A11 were detected by western blot. **C** Negative control siRNA or USP14 siRNA (50 nM) were transfected into cells. Before the cells were collected, the transfected cells were treated with CSE for 24 h, or combined with MG132 for 5 h. USP14, MFG-E8, GPx4, and SLC7A11 were detected by western blot. **D** Negative control siRNA or USP14 siRNA were (50 nM) transfected into cells. Before the cells were collected, the transfected cells were treated with CSE for 24 h, or pretreated with rhMFG-E8 for 2 h followed by CSE treatment for 24 h. USP14, MFG-E8, GPx4, and SLC7A11 were detected by western blot. Data are presented as the mean ± SD of three independent experiments. ^*^P < 0.05, compared between the marked groups. ^**^P < 0.01^,^ compared between the marked groups. ^***^P < 0.001, compared between the marked groups.

CSE diminished MFG-E8 protein level, but the exact mechanism was unclear. We stimulated the bronchial epithelial cells with CSE and/or MG132. The immunoblotting analysis showed that the proteasome inhibitor MG132 prevented MFG-E8 proteins from CSE-induced degradation (Fig. 9A), suggesting CSE downregulated MFG-E8 expression through the proteasome system.

To determine whether CSE-induced downregulation of MFG-E8 is mediated by the reduced USP14 expression, we modified USP14 with overexpressed plasmids and siRNA in BEAS-2B and HBE cells and treated cells with CSE. Overexpression of USP14 significantly inhibited the reduction in MFG-E8 protein induced by CSE (Fig. 9B). Silencing of USP14 promoted the protein reduction of MFG-E8 in CSE-treated cells (Fig. 9C). Furthermore, the decreased MFG-E8 level in USP14 silenced cells under CSE treatment could be rescued by MG132 (Fig. 9C). The above data suggested that CSE decreased USP14 to promote MFG-E8 proteasomal degradation, thus reduced MFG-E8 protein level in bronchial epithelial cells.

### USP14/MFG-E8 axis involves in CSE-induced ferroptosis in human bronchial epithelial cells

Combined with the above data, we hypothesized that CSE-induced ferroptosis of bronchial epithelial cells might be regulated by the USP14/MFG-E8 axis. We found that the CSE-regulated MFG-E8 level and its downstream signals were modulated by USP14. USP14 overexpression enhanced the promoting effects of MFG-E8 on GPx4 and SLC7A11 expressions in bronchial epithelial cells treated with CSE (Fig. 9B). Suppression of USP14 enhanced the reduction in GPx4 and SLC7A11 levels induced by CSE (Fig. 9C). MG132 could also significantly reverse the decreased levels of MFG-E8 downstream signals in USP14 silenced cells exposed to CSE (Fig. 9C).

To further test whether USP14 ameliorates ferroptosis through regulating MFG-E8 expression in CSE-exposed cells, USP14 siRNA and rhMFG-E8 were simultaneously applied to treat bronchial epithelial cells. The decreased levels of GPx4 and SLC7A11 induced by the depletion of USP14 could be rescued by the administration of rhMFG-E8 in CSE-treated cells (Fig. 9D). Collectively, these data indicated that USP14/MFG-E8 axis played an important role in regulating CSE-induced ferroptosis of human bronchial epithelial cells.

## Discussion

**Fig. 10:**
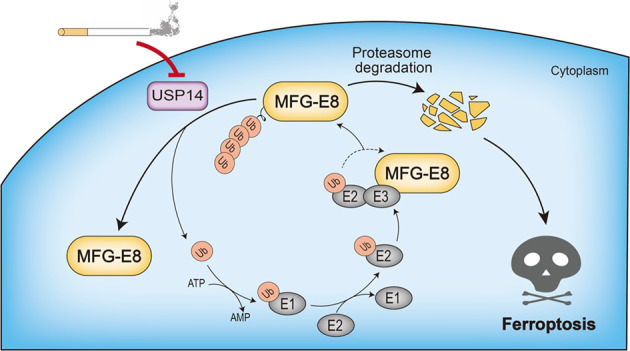
The inhibition of USP14/MFG-E8 axis induced by cigarette smoke aggravates ferroptosis in bronchial epithelial cells. MFG-E8 is dynamic at protein level that is stringently monitored by the ubiquitin–proteasome system. USP14 interacts with, deubiquitinates, and stabilizes MFG-E8. Cigarette smoke exposure diminishes USP14 expression. Depletion of USP14 leads to the proteasomal degradation of MFG-E8, which aggravates bronchial epithelial cell ferroptosis.

To our knowledge, this study provided the first evidence that (1) MFG-E8 protein level was downregulated in bronchial epithelial cells of COPD patients and CSE-induced emphysematous mice, and CSE decreased MFG-E8 protein in human bronchial epithelial cells in vitro; (2) MFG-E8 effectively inhibited ferroptosis induced by CSE in vivo and in vitro; (3) Cigarette smoke showed no significant effect on MFG-E8 mRNA levels in lung tissues of humans and mice and in bronchial epithelial cell lines; (4) USP14 was a specific cytoplasmic DUB for MFG-E8 deubiquitination; (5) CSE could decrease USP14 expression to downregulate MFG-E8 via enhancing MFG-E8 proteasomal degradation; and (6) USP14/MFG-E8 axis involves in CSE-induced ferroptosis in human bronchial epithelial cells (Fig. 10).

Previous studies have demonstrated the antioxidant activity of MFG-E8. Li et al. reported that MFG-E8 reversed the elevation of lipid peroxidation product malondialdehyde (MDA) and ROS, and reduction of GSH and superoxide dismutase (SOD) induced by lipopolysaccharide (LPS) in mouse brains and primary microglia [23]. In mice with pristane-induced lupus, MFG-E8 knockout stimulated the overproduction of neutrophil extracellular traps, which were formed depending on the generation of ROS by nicotinamide adenine dinucleotide phosphate (NADPH) oxidase [20]. We further found that MFG-E8 could alleviate iron-dependent lipid peroxidation and cell damage. MFG-E8 is usually considered to be a secreted protein that exhibits its effects by binding to integrins on the cell membrane and then transducing downstream signals [9]. Fujiwara et al. proposed that angiosarcoma-derived MFG-E8 might enhance tumor growth in autocrine/paracrine manner [24]. In fact, MFG-E8 is widely distributed in cytosol, endoplasmic reticulum, and plasma membrane, according to the Human Protein Atlas database (http://www.proteinatlas.org/) and the GeneCards database (https://www.genecards.org/). In our study, IHC and IF both showed that MFG-E8 was located in the cytoplasm. MFG-E8 post-translational degradation in cytosol might decrease its secretion and affect the following regulation of downstream signal molecules.

MFG-E8 exhibited as a primary factor affecting ferroptosis and played an important role in resisting the harmful effects of CSE on human bronchial epithelial cells. A better comprehension of the MFG-E8 upstream regulatory mechanism might reveal new therapeutic targets for COPD patients. Ample evidence demonstrates that abnormal ubiquitination and disordered deubiquitination are extensively associated with COPD. Kim et al. reported that CSE induced Akt ubiquitination and proteasomal degradation through promoting ubiquitin E3 ligase expression, which led to cell death in normal human lung fibroblasts [25]. Recently, Li et al. found that F-box protein FBXW17, an E3 ubiquitin ligase, exaggerated CSE-induced lung epithelial inflammation and apoptosis via mediating the proteasomal degradation of protein methyltransferase PRMT6 [3]. However, the expression of deubiquitinase USP17 in CSE-exposed airway epithelial cells was downregulated, resulting in the degradation of histone deacetylase HDAC2 by ubiquitination and glucocorticoid resistance in COPD [26]. The ubiquitination and deubiquitination of some crucial proteins in lung epithelial cells can determine cell fate. For the first time, we proposed that MFG-E8 was a short-life protein and could be degraded in an ubiquitin–proteasome pathway. Moreover, CSE-induced MFG-E8 protein reduction was partly rescued by the proteasome inhibitor MG132, thus influencing the expression of ferroptosis-related factors.

Deubiquitinase USP14 is known to be extensively engaged in varying canonical signaling pathways, including the NF-κB and Wnt/β-catenin pathways [27]. In addition, USP14 inhibits the autophagic occurrence by suppressing ubiquitination of autophagy regulator Beclin 1 [28]. A recent study found that USP14 stabilized Beclin 1 to reduce autophagy-dependent ferroptosis induced by 6-Gingerol in lung cancer cells [29]. Nevertheless, the signal transduction of USP14 upon cigarette smoke exposure has not been elucidated. In this study, we showed that USP14 was decreased in CSE-exposed human bronchial epithelial cells. USP14 regulated MFG-E8 post-translationally by the inhibition of MFG-E8 ubiquitination. Moreover, USP14 inhibited CSE-induced MFG-E8 proteasomal degradation, thus protecting bronchial epithelial cells from ferroptosis. Our study highlighted the importance of manipulating the proteolytic processing of MFG-E8 by a DUB, which was suggested to profoundly attenuate the harmful effects of CSE. Our research also added a paradigm of USP14 function in cell fate decisions after CSE exposure.

In our study, the mechanism by which MFG-E8 regulated CSE-induced ferroptosis required further investigation. The cystine/glutamate antiporter containing SLC7A11 is a transmembrane protein complex. MFG-E8 might activate SLC7A11 directly or regulate SLC7A11 via integrin pathway. Additionally, the role of USP14 and its effects on MFG-E8 protein levels were not studied in animal lung tissues exposed to cigarette smoke. However, we conducted relevant experiments in two human bronchial epithelial cell lines at the same time, which could enhance the confidence of our data.

In conclusion, this study demonstrated that cigarette smoke diminished MFG-E8 protein level but had no significant effect on its mRNA level in bronchial epithelial cells. MFG-E8 could attenuate CSE-induced ferroptosis in vivo and in vitro. Moreover, USP14 interacted with, deubiquitinated and stabilized MFG-E8. USP14 downregulated by CSE-induced MFG-E8 proteasomal degradation and further reduced the antiferroptotic effect of MFG-E8. These results elucidate a novel mechanism of MFG-E8 regulation and demonstrate the antiferroptotic activity of MFG-E8 for the first time. Our findings also provide insight into the potential role of USP14 in the pathogenesis of COPD.

## Supplementary information

Supplementary Material

Reproducibility checklist

Original Data File

## Data Availability

The raw data acquired for this study are available from the corresponding author upon reasonable request.

## References

[1] Committee GE. Global strategy for the diagnosis, management and prevention of chronic obstructive pulmonary disease (2022 report). 2021. Accessed Nov 2021 at http://goldcopd.com.

[2] Li T, Fanning KV, Nyunoya T, Chen Y, Zou C (2020). Cigarette smoke extract induces airway epithelial cell death via repressing prmt6/akt signaling. Aging.

[3] Li T, He X, Luo L, Zeng H, Ren S, Chen Y (2021). F-box protein fbxw17-mediated proteasomal degradation of protein methyltransferase prmt6 exaggerates cse-induced lung epithelial inflammation and apoptosis. Front Cell Dev Biol.

[4] Dixon SJ, Lemberg KM, Lamprecht MR, Skouta R, Zaitsev EM, Gleason CE (2012). Ferroptosis: an iron-dependent form of nonapoptotic cell death. Cell..

[5] Hirschhorn T, Stockwell BR (2019). The development of the concept of ferroptosis. Free Radic Biol Med.

[6] Jiang X, Stockwell BR, Conrad M (2021). Ferroptosis: mechanisms, biology and role in disease. Nat Rev Mol Cell Biol.

[7] Yoshida M, Minagawa S, Araya J, Sakamoto T, Hara H, Tsubouchi K (2019). Involvement of cigarette smoke-induced epithelial cell ferroptosis in COPD pathogenesis. Nat Commun.

[8] Liu X, Ma Y, Luo L, Zong D, Li H, Zeng Z (2022). Dihydroquercetin suppresses cigarette smoke induced ferroptosis in the pathogenesis of chronic obstructive pulmonary disease by activating nrf2-mediated pathway. Phytomedicine..

[9] Yi YS (2016). Functional role of milk fat globule-epidermal growth factor viii in macrophage-mediated inflammatory responses and inflammatory/autoimmune diseases. Mediators Inflamm.

[10] Huang W, Jiao J, Liu J, Huang M, Hu Y, Ran W (2020). Mfg-e8 accelerates wound healing in diabetes by regulating “nlrp3 inflammasome-neutrophil extracellular traps” axis. Cell Death Discov.

[11] Bu HF, Zuo XL, Wang X, Ensslin MA, Koti V, Hsueh W (2007). Milk fat globule-EGF factor 8/lactadherin plays a crucial role in maintenance and repair of murine intestinal epithelium. J Clin Invest.

[12] Wang M, Fu Z, Wu J, Zhang J, Jiang L, Khazan B (2012). Mfg-e8 activates proliferation of vascular smooth muscle cells via integrin signaling. Aging Cell.

[13] Zhang S, Xie JG, Su BT, Li JL, Hu N, Chen J (2015). Mfg-e8, a clearance glycoprotein of apoptotic cells, as a new marker of disease severity in chronic obstructive pulmonary disease. Braz J Med Biol Res.

[14] Wang Y, Luo G, Chen J, Jiang R, Zhu J, Hu N (2017). Cigarette smoke attenuates phagocytic ability of macrophages through down-regulating milk fat globule-EGF factor 8 (mfg-e8) expressions. Sci Rep..

[15] Schwartz AL, Ciechanover A (2009). Targeting proteins for destruction by the ubiquitin system: implications for human pathobiology. Annu Rev Pharm Toxicol.

[16] Shen M, Schmitt S, Buac D, Dou QP (2013). Targeting the ubiquitin-proteasome system for cancer therapy. Expert Opin Ther Targets.

[17] Stintzing S, Lenz HJ (2014). Molecular pathways: turning proteasomal protein degradation into a unique treatment approach. Clin Cancer Res.

[18] Fraile JM, Quesada V, Rodríguez D, Freije JM, López-Otín C (2012). Deubiquitinases in cancer: new functions and therapeutic options. Oncogene..

[19] Chen L, Luo L, Kang N, He X, Li T, Chen Y (2020). The protective effect of hbo1 on cigarette smoke extract-induced apoptosis in airway epithelial cells. Int J Chronic Obstr Pulm Dis.

[20] Huang W, Wu J, Yang H, Xiong Y, Jiang R, Cui T (2017). Milk fat globule-EGF factor 8 suppresses the aberrant immune response of systemic lupus erythematosus-derived neutrophils and associated tissue damage. Cell Death Differ.

[21] Wang J, Wu J, Zhu X, Chen J, Zhao J, Xu Y (2021). Absence of the mfg-e8 gene prevents hypoxia-induced pulmonary hypertension in mice. J Cell Physiol.

[22] He Z-H, Chen P, Chen Y, He S-D, Ye J-R, Zhang H-L (2015). Comparison between cigarette smoke-induced emphysema and cigarette smoke extract-induced emphysema. Tob Induc Dis.

[23] Li J, Xu X, Cai X, Weng Y, Wang Y, Shen Q (2019). Milk fat globule-epidermal growth factor-factor 8 reverses lipopolysaccharide-induced microglial oxidative stress. Oxid Med Cell Longev.

[24] Fujiwara C, Motegi S-I, Ohira A, Yamaguchi S, Sekiguchi A, Yasuda M (2019). The significance of tumor cells-derived mfg-e8 in tumor growth of angiosarcoma. J Dermatol Sci.

[25] Kim S-Y, Lee J-H, Huh JW, Ro JY, Oh Y-M, Lee S-D (2011). Cigarette smoke induces Akt protein degradation by the ubiquitin-proteasome system. J Biol Chem.

[26] Song H, Tao L, Chen C, Pan L, Hao J, Ni Y (2015). Usp17-mediated deubiquitination and stabilization of hdac2 in cigarette smoke extract-induced inflammation. Int J Clin Exp Pathol.

[27] Wang F, Ning S, Yu B, Wang Y (2021). Usp14: structure, function, and target inhibition. Front Pharm.

[28] Xu D, Shan B, Sun H, Xiao J, Zhu K, Xie X (2016). Usp14 regulates autophagy by suppressing k63 ubiquitination of beclin 1. Genes Dev.

[29] Tsai Y, Xia C, Sun Z (2020). The inhibitory effect of 6-gingerol on ubiquitin-specific peptidase 14 enhances autophagy-dependent ferroptosis and anti-tumor in vivo and in vitro. Front Pharm.

