# Supplementary material for: MFG-E8 stabilized by deubiquitinase USP14 suppresses cigarette smoke-induced ferroptosis in bronchial epithelial cells
Source: Cell Death Dis. 2023 Jan 3;14(1):2. doi: 10.1038/s41419-022-05455-8 (PMC9810602; doi:10.1038/s41419-022-05455-8)
Supplement: Supplementary file 1 — Supplementary Material [file 41419_2022_5455_MOESM1_ESM.docx]

**Supplementary Material**

**Table S1 Sequences of primers used for MFG‐E8 gene identification in mice**

| **Gene** | **Forward primer** | **Reverse primer** |
| --- | --- | --- |
| MFG-E8 KO | 5’-GTGGGCAAGTGCATCTGAGTAC-3’ | 5’-GAGCGATCCTATCTCAAAACCAA-3’ |
| MFG-E8 WT | 5’-TTGCCAACAGGCTTGATGGATAT-3’ | 5’-GACAGTACGGAACAGCGAAGGTA-3’ |

**Table S2 shRNA sequences**

| **Gene** | **shRNA sequence** |
| --- | --- |
| MFG-E8 | 5’-ACCCAGCAGCAATGACGATAA-3’ |
| Non-targeting control | 5’-TTCTCCGAACGTGTCACGT-3’ |

**Table S3 siRNA sequence**

| **Gene** | **siRNA sequence** |
| --- | --- |
| USP14 | 5’-TTGCCGAGAAAGGTGAACA-3’ |

**Table S4 Sequences of primers used for RT-qPCR**

| **Gene** | **Forward primer** | **Reverse primer** |
| --- | --- | --- |
| hsa-MFG-E8 | 5’-CCTGCCACAACGGTGGTTTAT‐3’ | 5’‐GCGATCTGTGAGTTGGCAATGT‐3’ |
| has-USP14 | 5’-GGGAAATGGCTTCAGCGCAGTA‐3’ | 5’-CACCTTTCTCGGCAAACTGTGG‐3’ |
| has-GPx4 | 5’-ACAAGAACGGCTGCGTGGTGAA-3’ | 5’- GCCACACACTTGTGGAGCTAGA-3’ |
| has-SLC7A11 | 5’-TCCTGCTTTGGCTCCATGAACG-3’ | 5’-AGAGGAGTGTGCTTGCGGACAT-3’ |
| has-GAPDH | 5’-GTCTCCTCTGACTTCAACAGCG-3’ | 5’-ACCACCCTGTTGCTGTAGCCAA-3’ |
| mmu-MFG-E8 | 5’-TTGCCAACAGGCTTGATGGATAT‐3’ | 5’-GACAGTACGGAACAGCGAAGGTA‐3’ |
| mmu-GPx4 | 5’-CCTCTGCTGCAAGAGCCTCCC-3’ | 5’-CTTATCCAGGCAGACCATGTGC-3’ |
| mmu-SLC7A11 | 5’-CTTTGTTGCCCTCTCCTGCTTC-3’ | 5’-CAGAGGAGTGTGCTTGTGGACA-3’ |
| mmu-GAPDH | 5’-CATCACTGCCACCCAGAAGACTG-3’ | 5’-ATGCCAGTGAGCTTCCCGTTCAG-3’ |

**
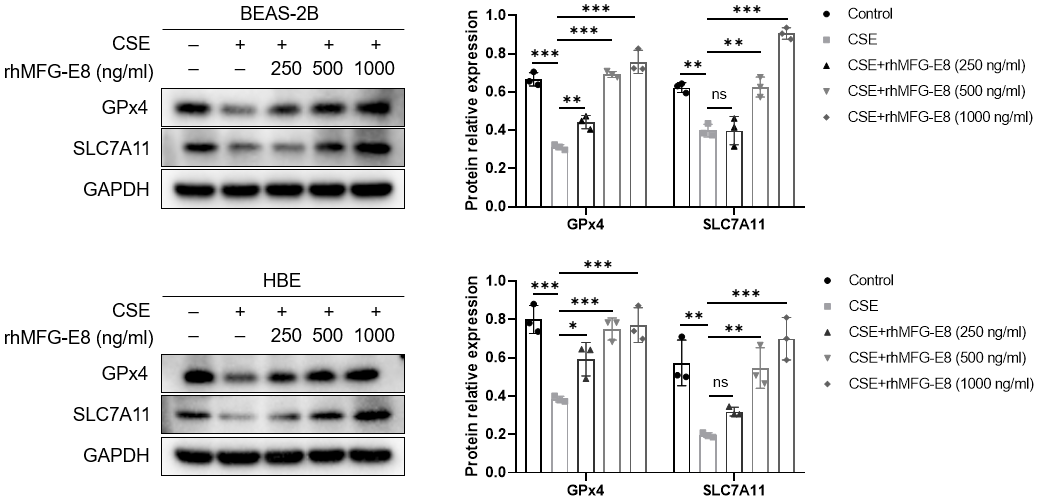
**

**Figure S1** Effects of different concentrations of rhMFG-E8 on ferroptosis induced by CSE in BEAS-2B cells and HBE cells. Ferroptosis related proteins (GPx4 and SLC7A11) were detected by Western blot. Data are presented as the mean ± SD of three independent experiments. ^*^P < 0.05, compared between the marked groups. ^**^P < 0.01, compared between the marked groups. ^***^P < 0.001, compared between the marked groups.

**
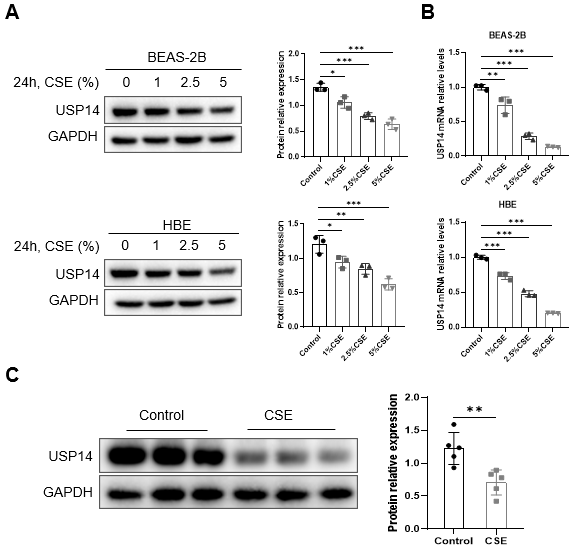
**

**Figure S2** CSE exposure diminishes USP14 expression in vivo and in vitro. (A) Western blot analyses of USP14 protein levels in BEAS-2B cells and HBE cells exposed to CSE. (B) Levels of USP14 mRNA in BEAS-2B cells and HBE cells exposed to CSE. (C) Western blot analyses of USP14 protein levels in lung tissues of mice. Control: WT mice exposed to PBS. CSE: WT mice exposed to CSE. Data are presented as the mean ± SD of three independent experiments. ^*^P < 0.05, compared between the marked groups. ^**^P < 0.01, compared between the marked groups. ^***^P < 0.001, compared between the marked groups.
