# Supplementary material for: MFG-E8 stabilized by deubiquitinase USP14 suppresses cigarette smoke-induced ferroptosis in bronchial epithelial cells
Source: Cell Death Dis. 2023 Jan 3;14(1):2. doi: 10.1038/s41419-022-05455-8 (PMC9810602; doi:10.1038/s41419-022-05455-8)

Figure 1C

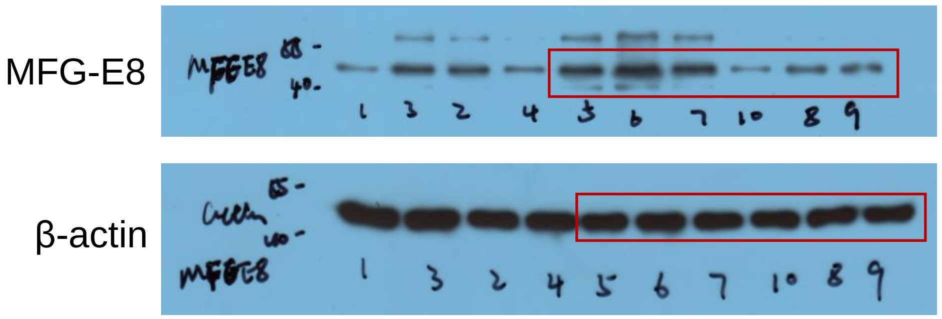

Figure 1D

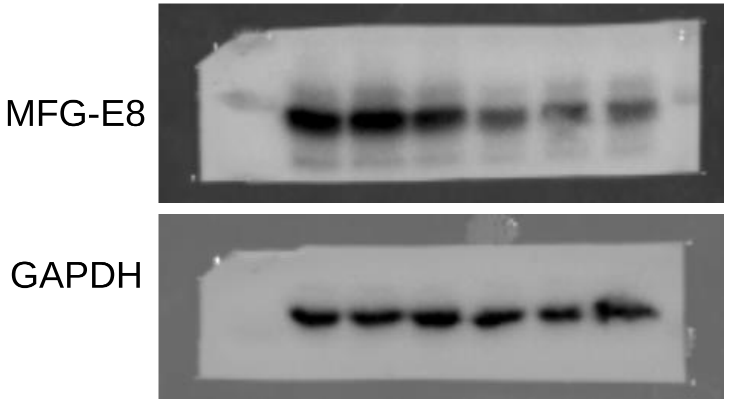

Figure 1E

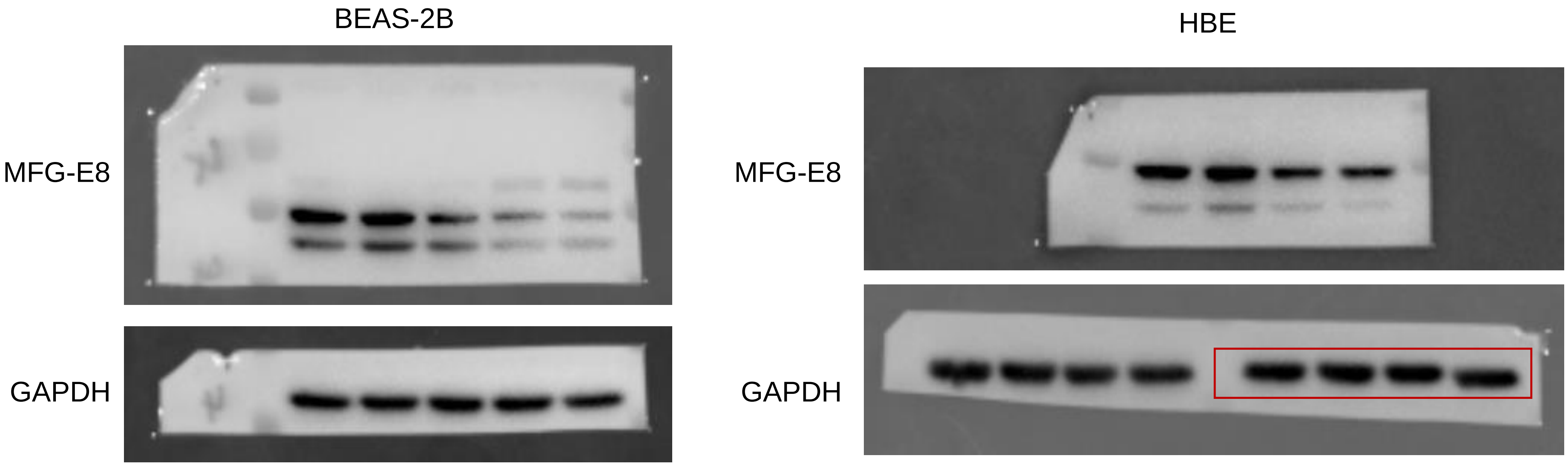

Figure 2B

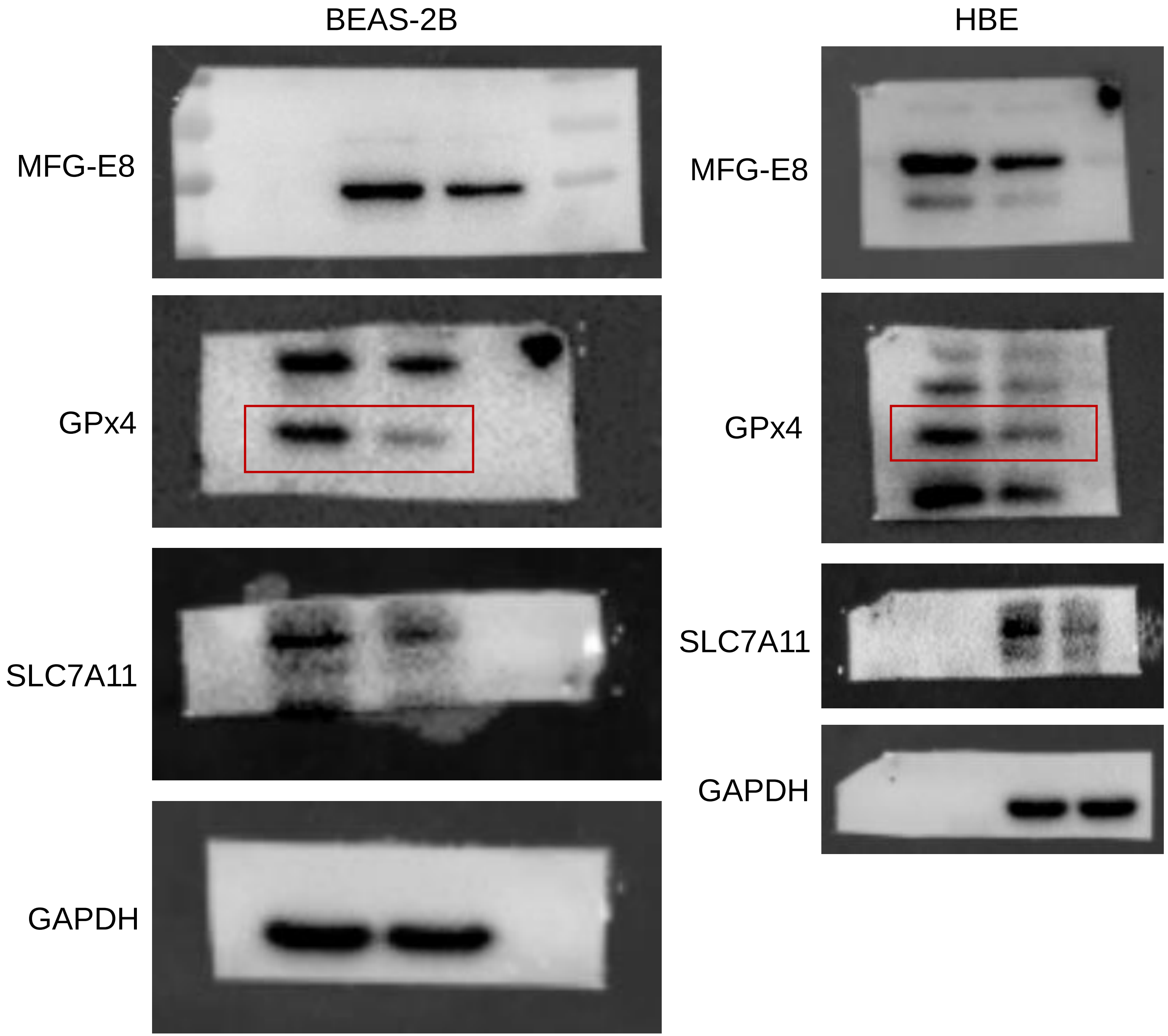

Figure 3B

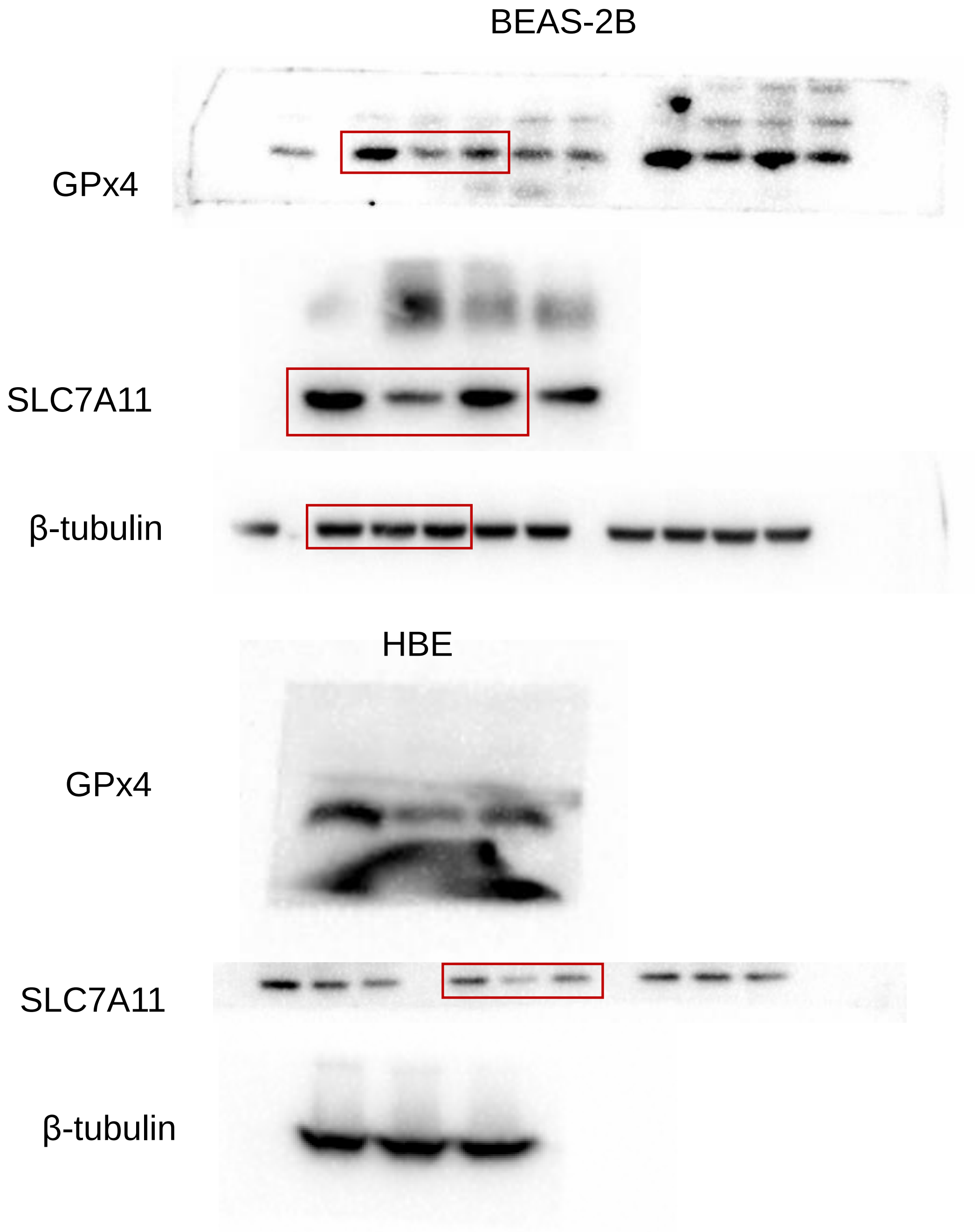

Figure 4H

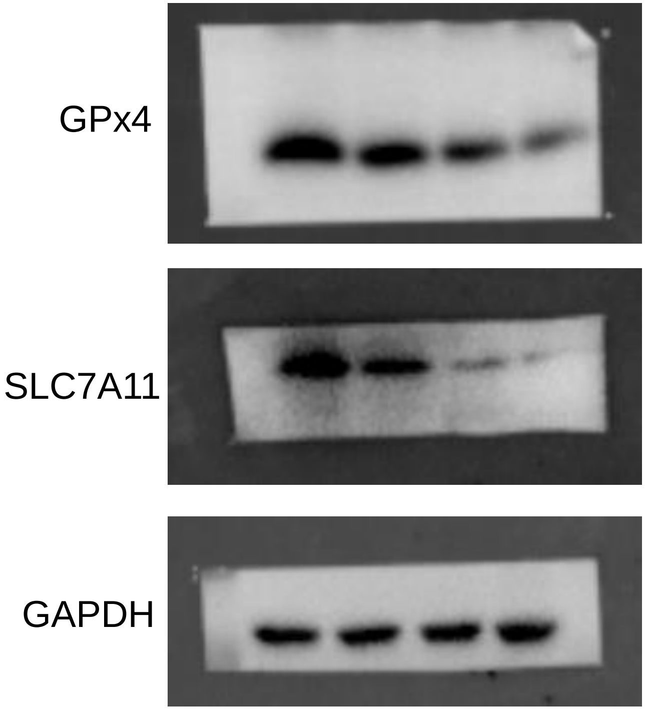

Figure 6A

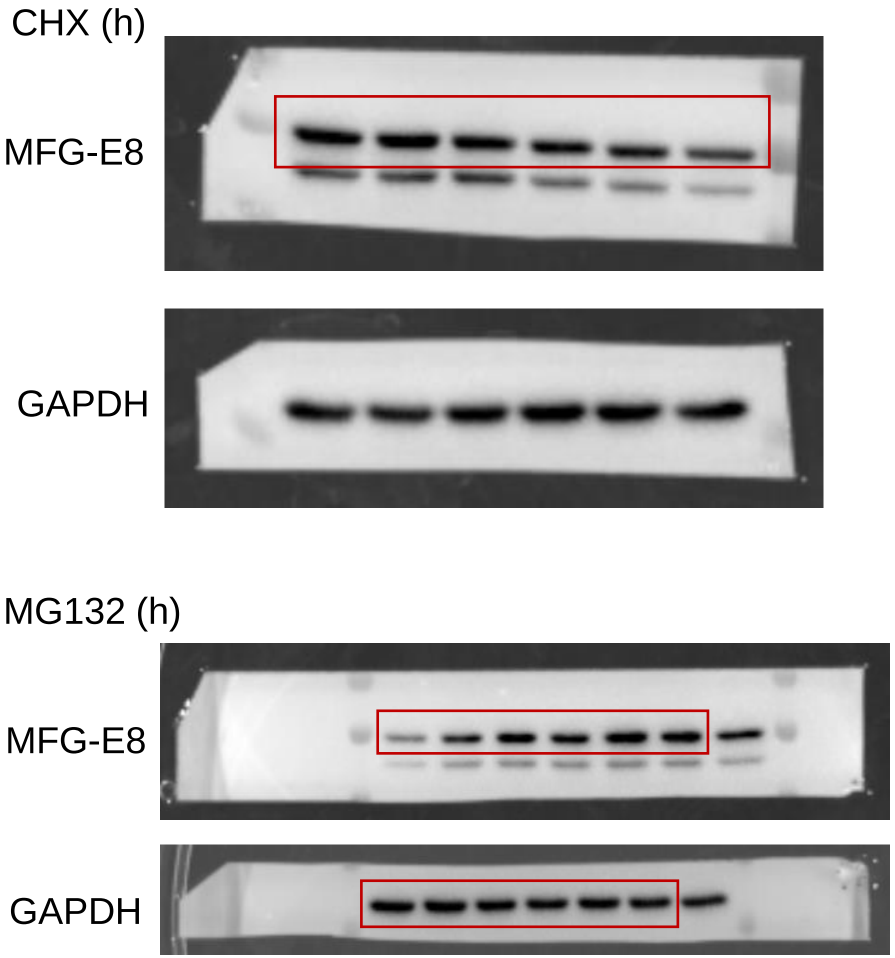

Figure 6C

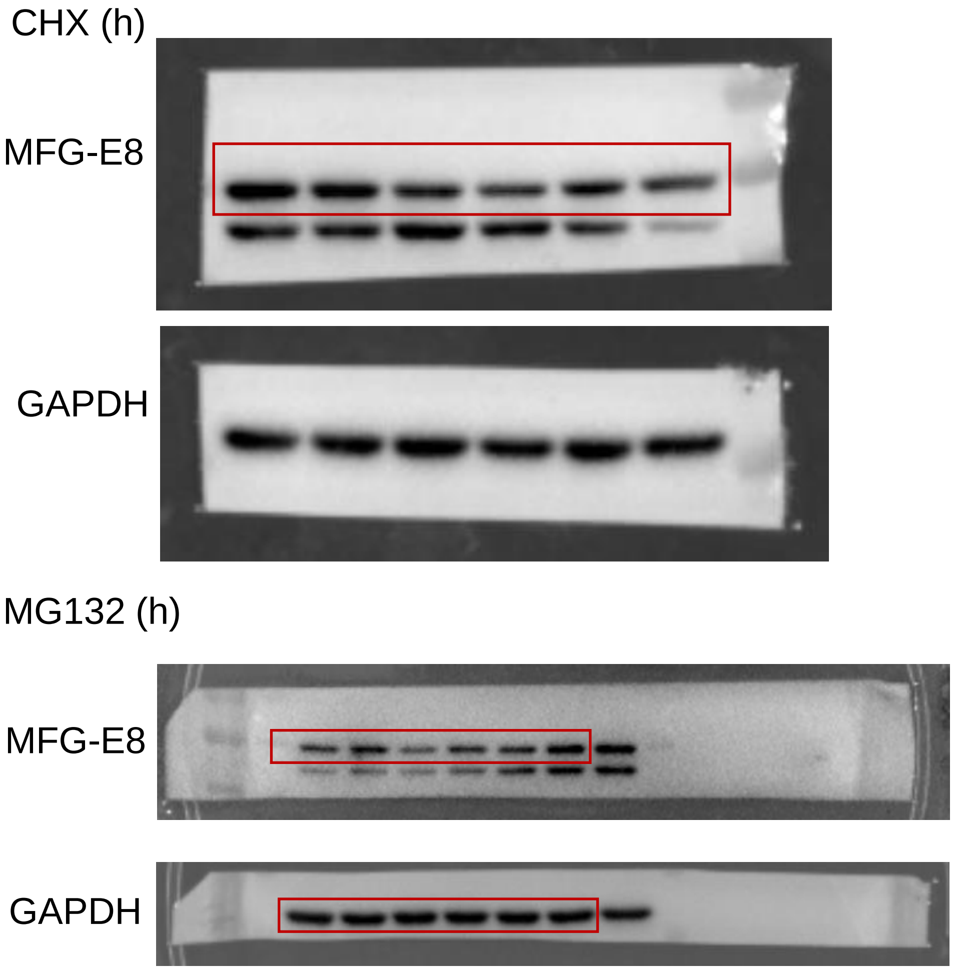

Figure 7A

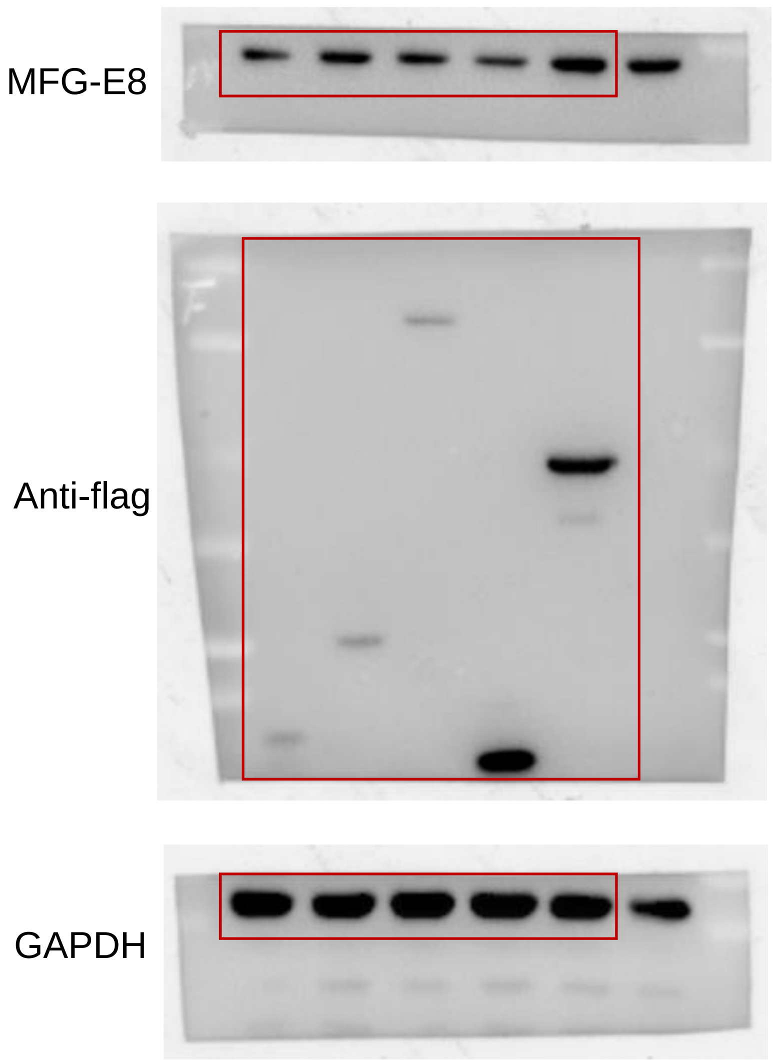

Figure 7B

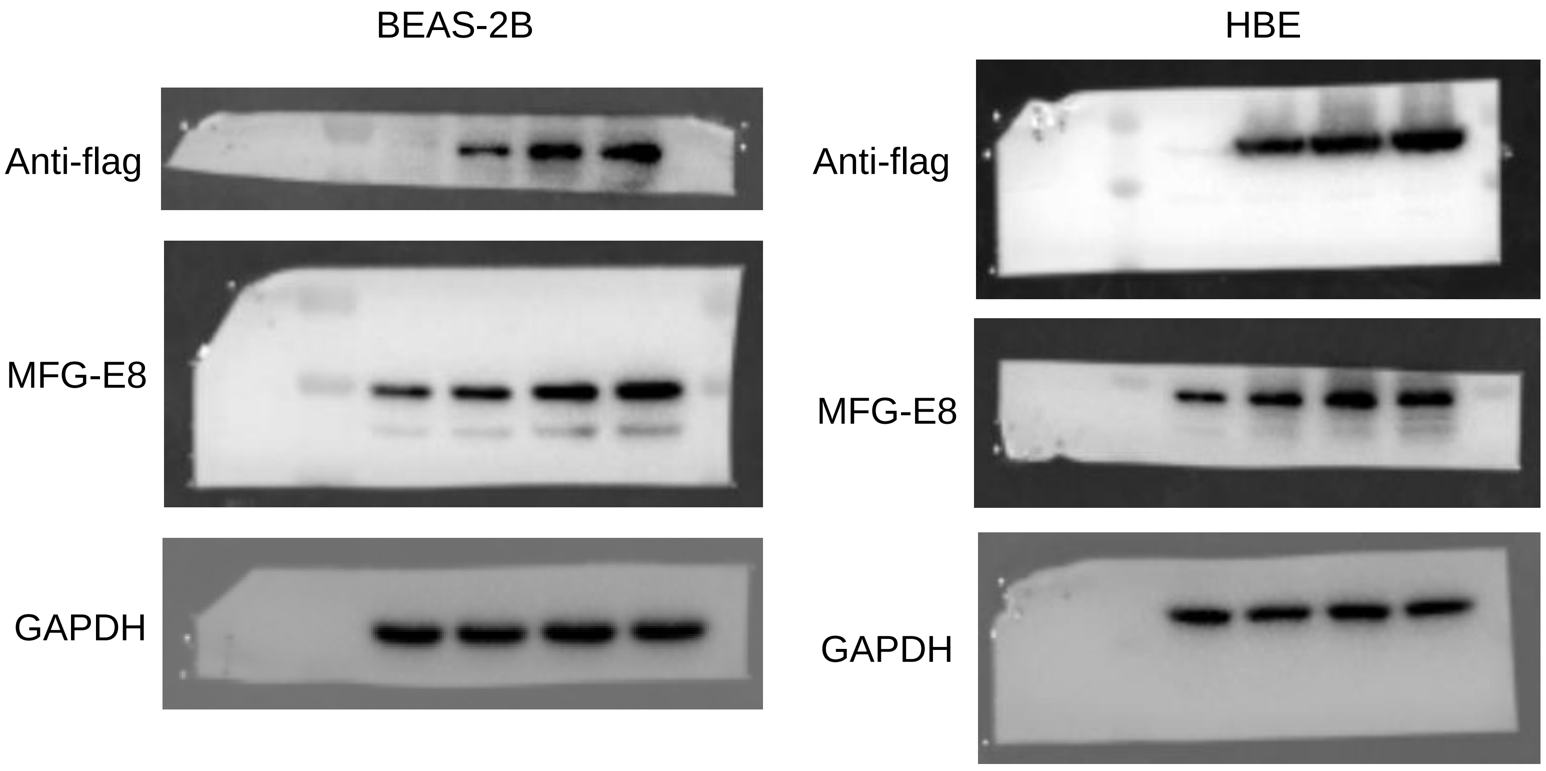

Figure 7C

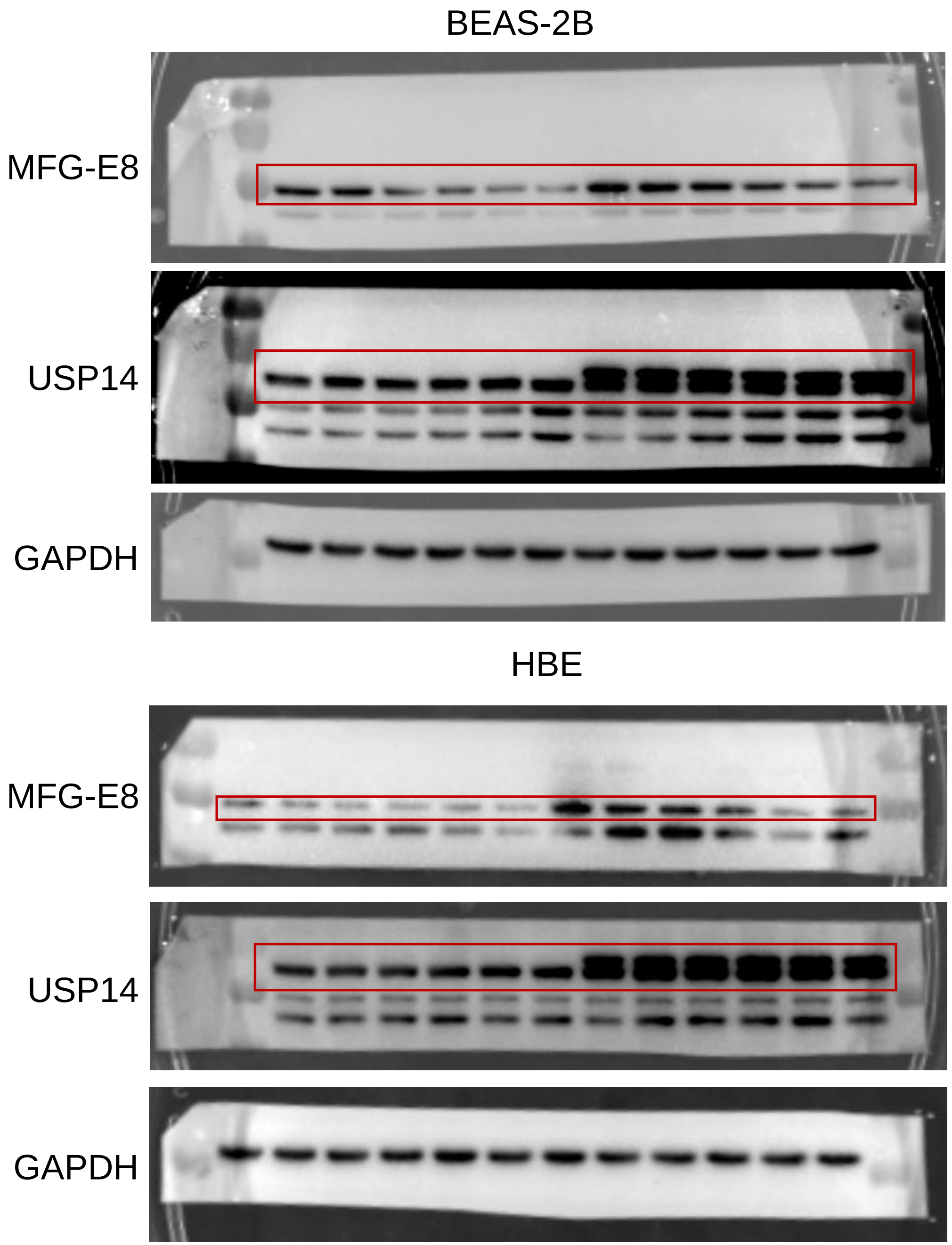

Figure 7D

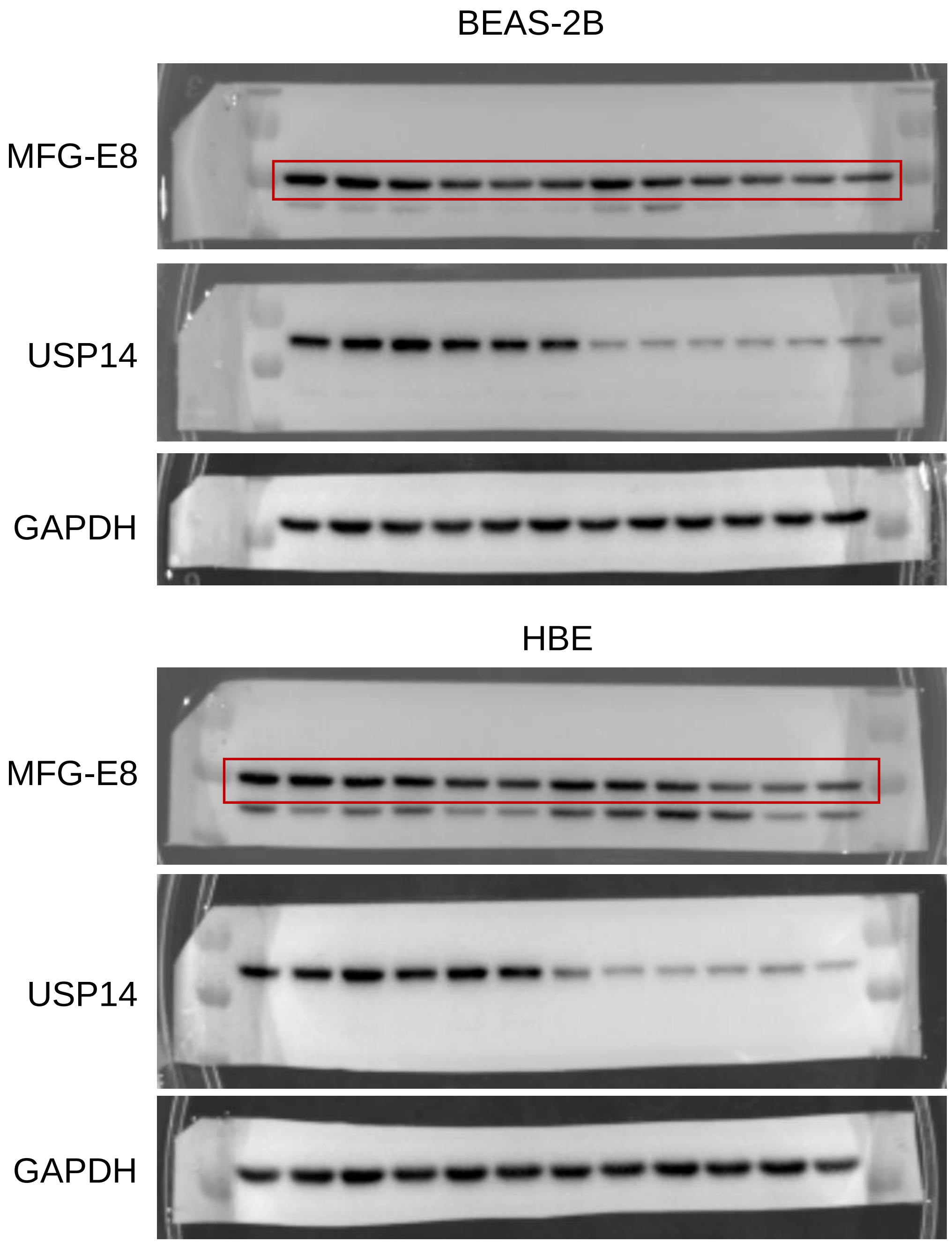

Figure 8B

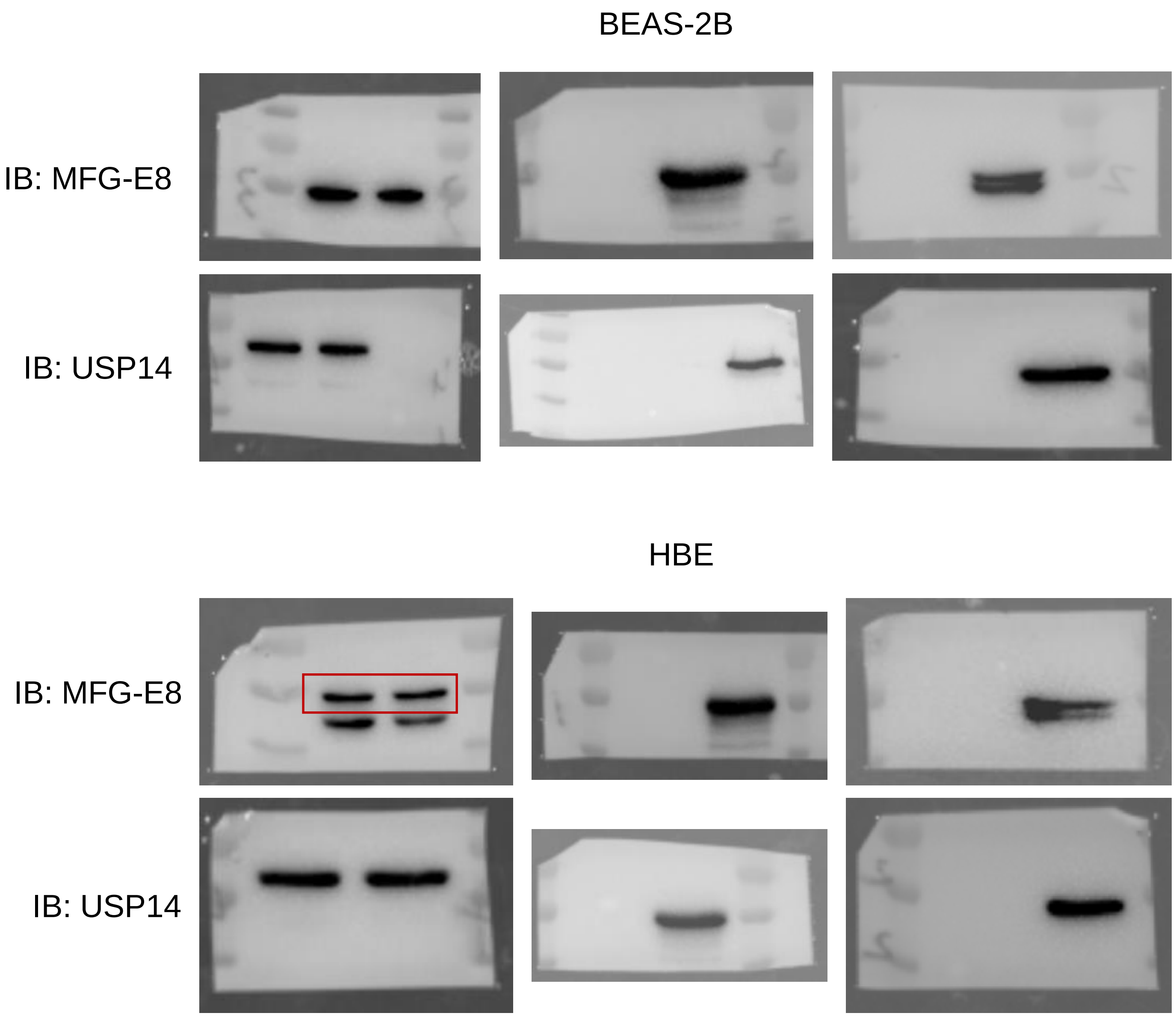

Figure 8C

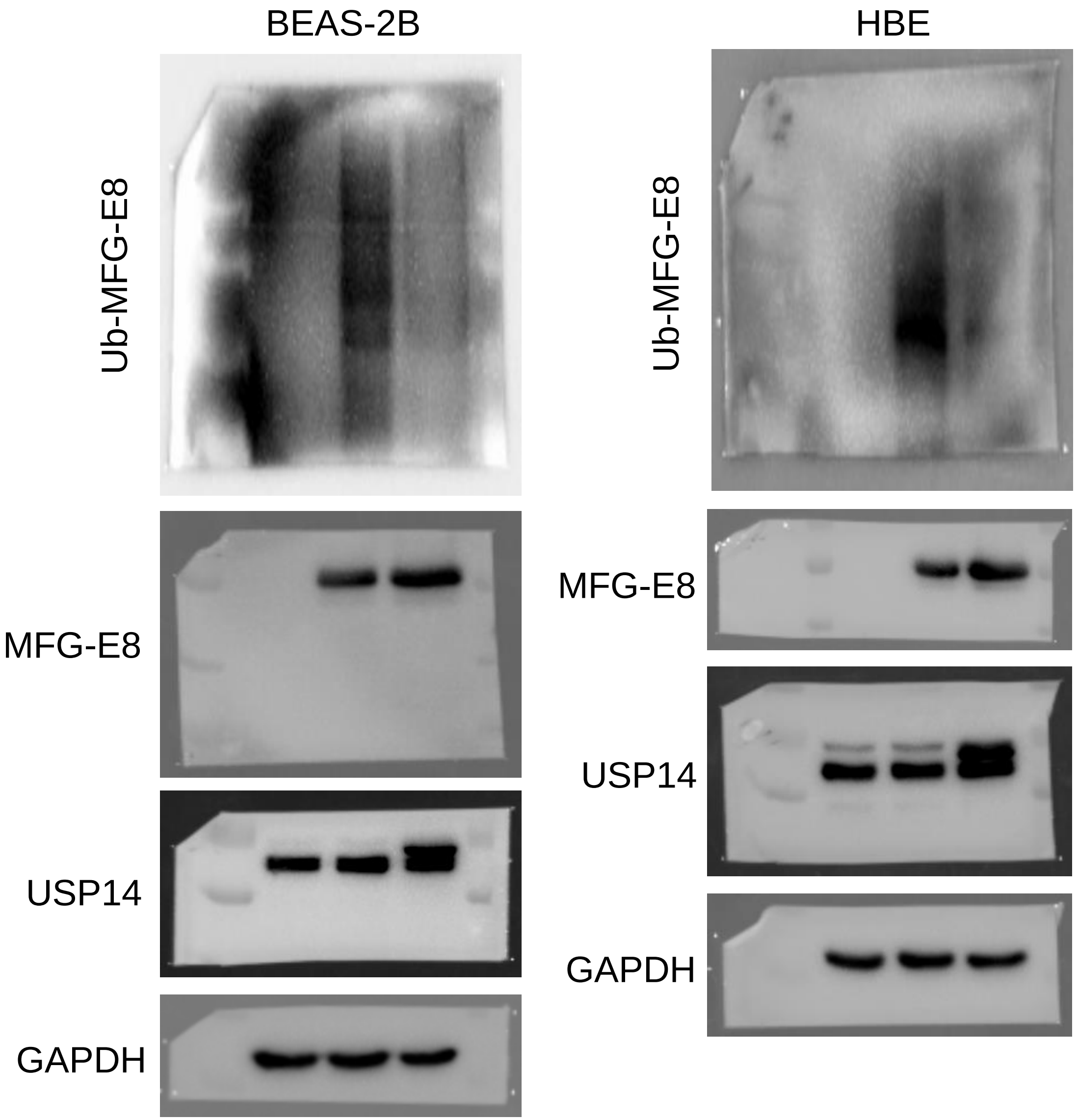

Figure 8D

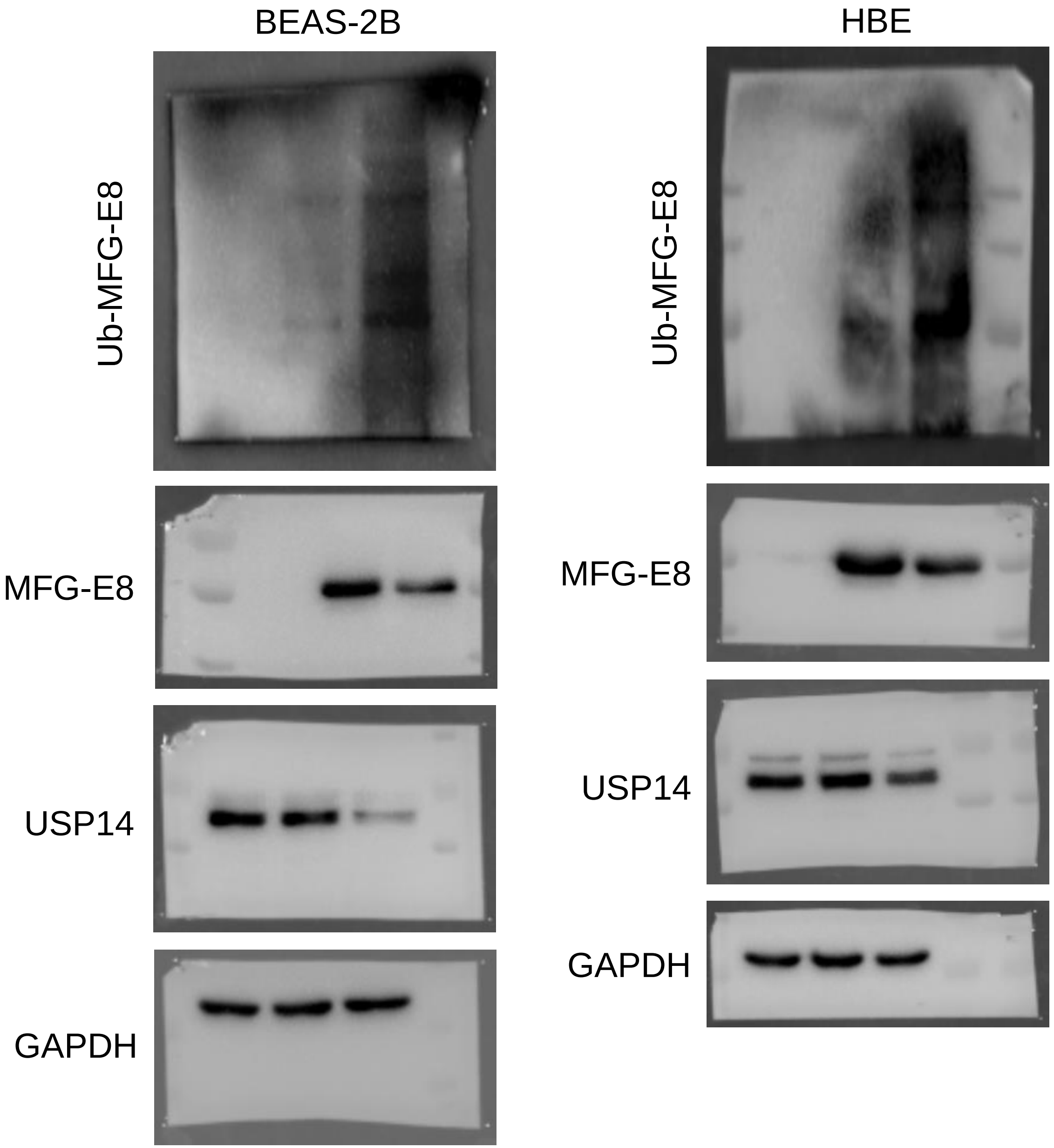

Figure 9A

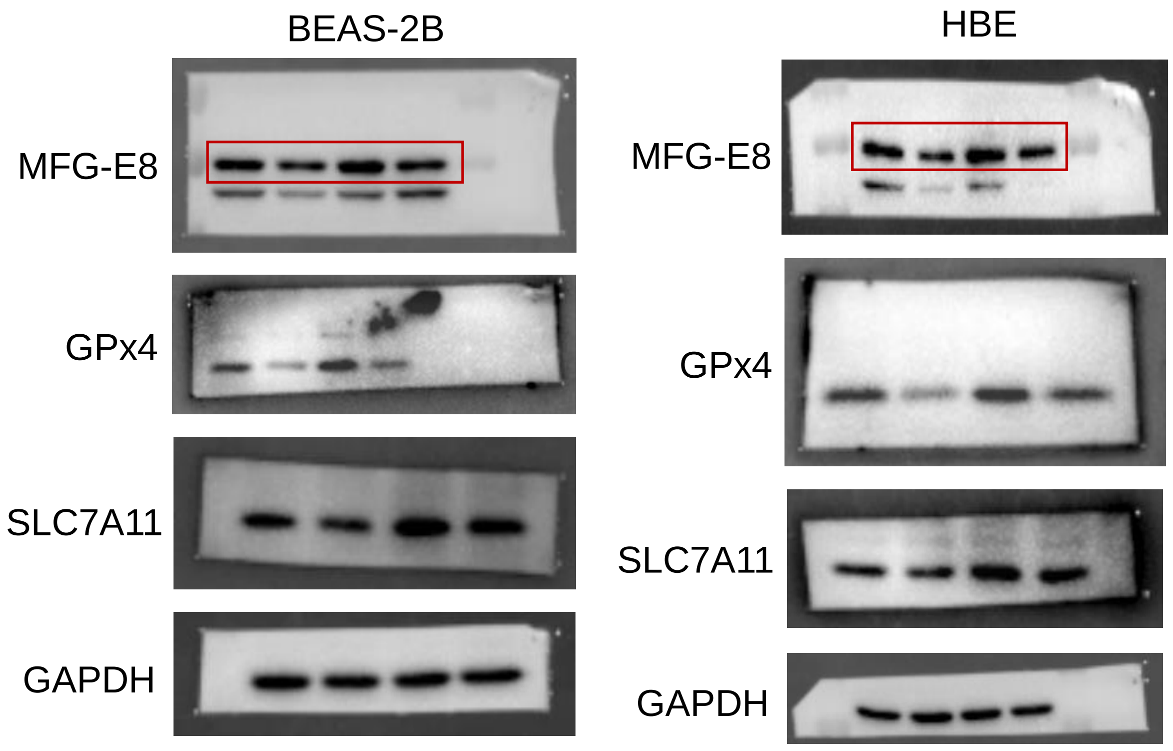

Figure 9B

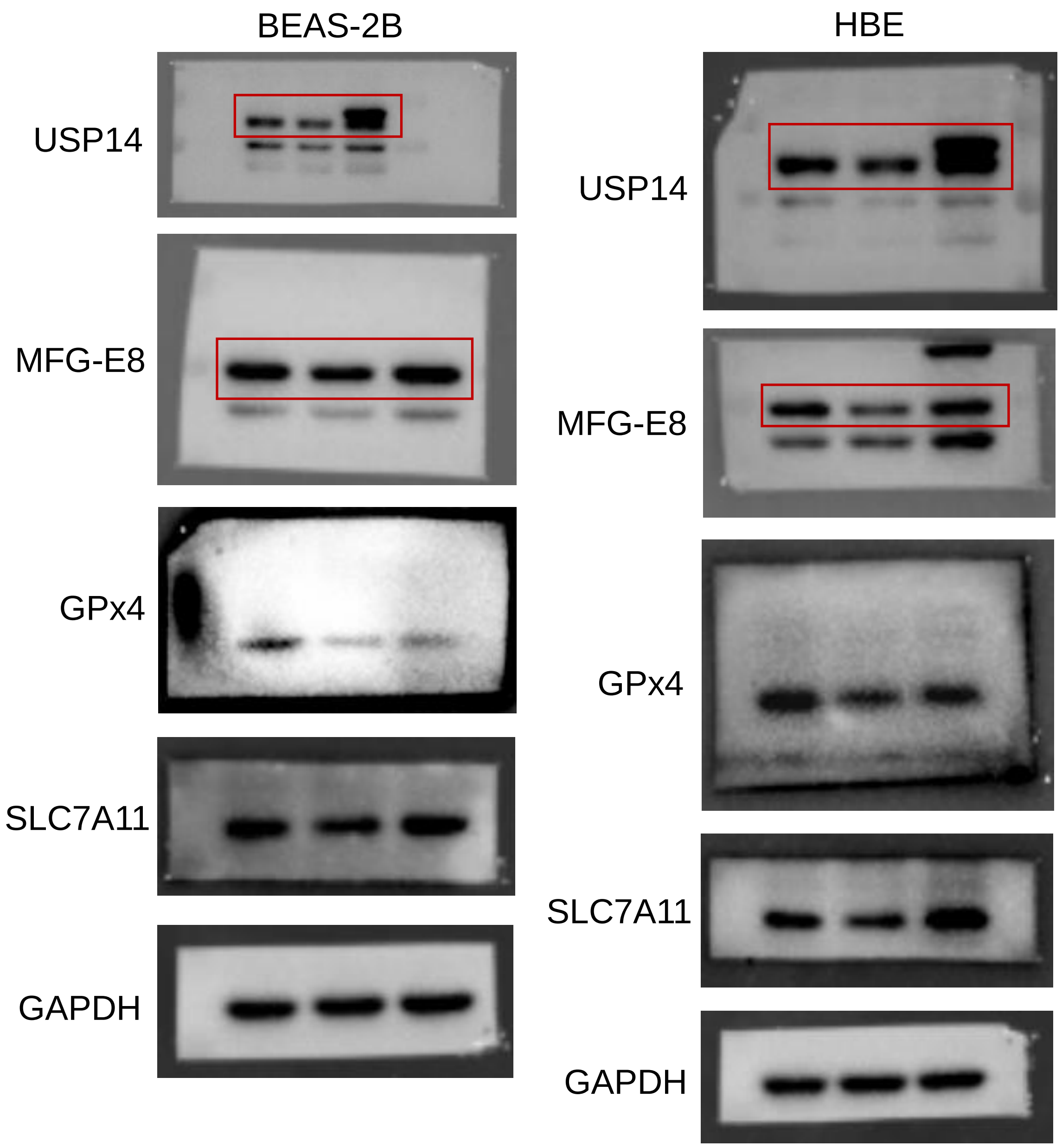

Figure 9C

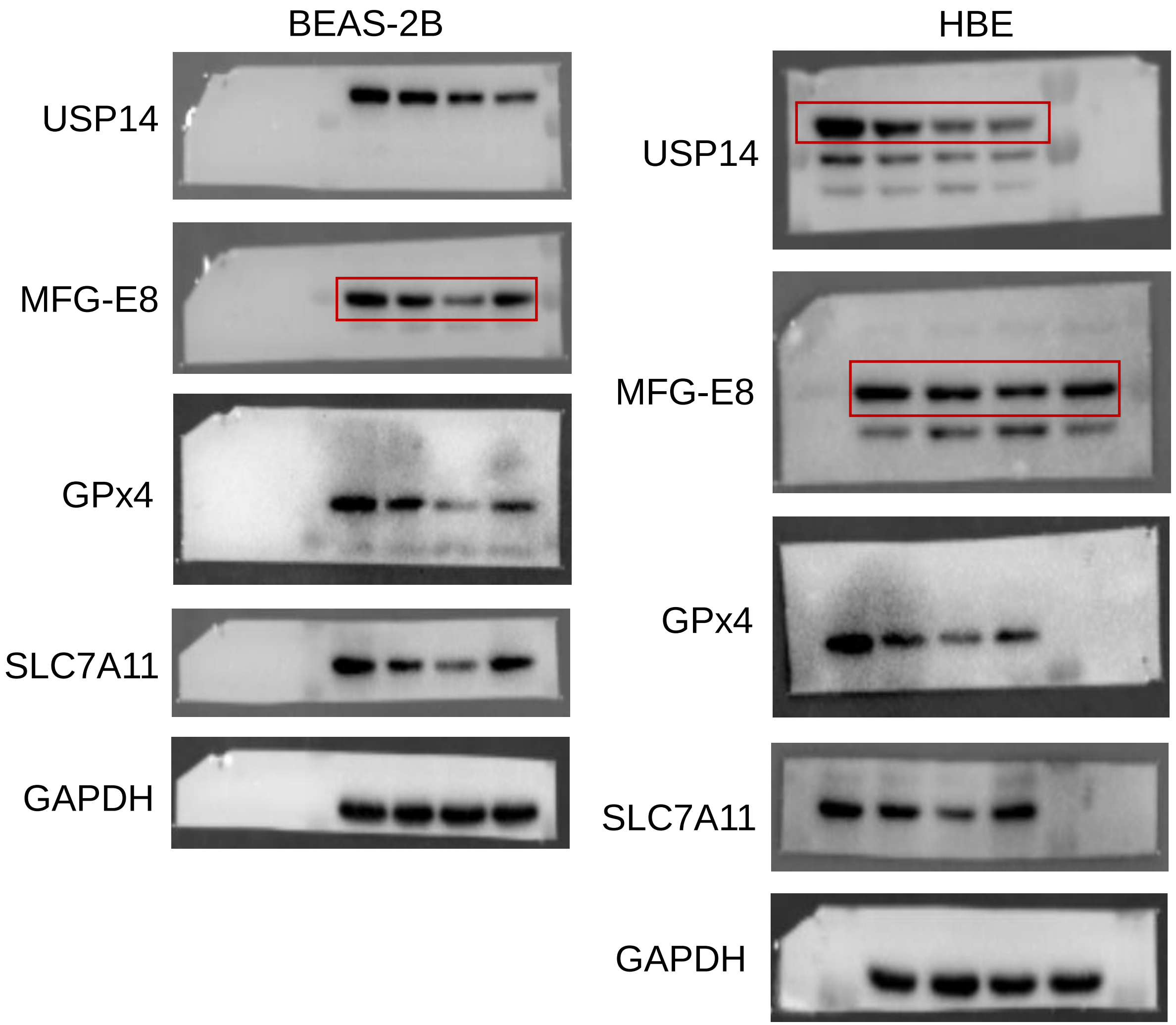

Figure 9D

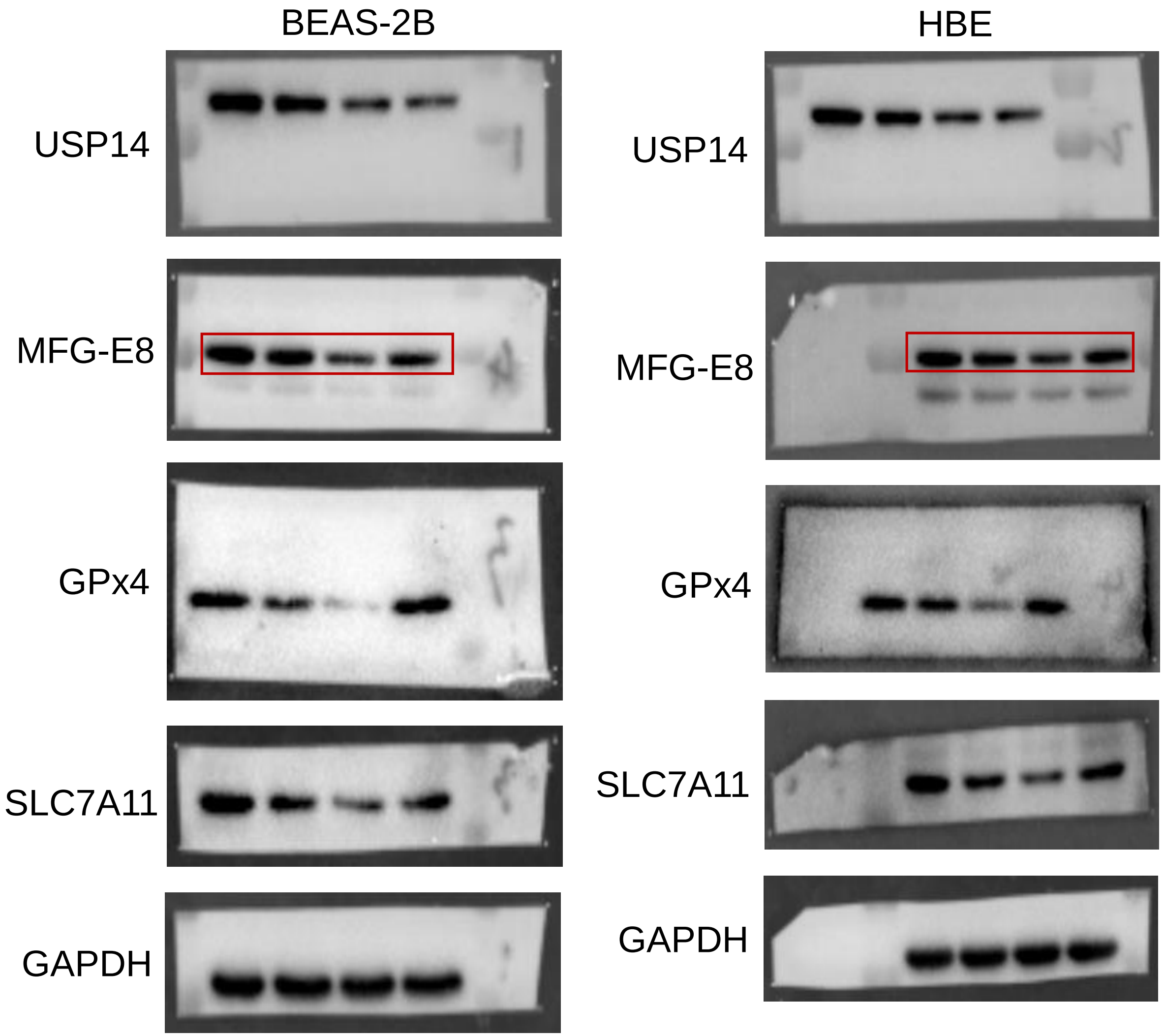

Supplement: Supplementary file 3 — Original Data File [file 41419_2022_5455_MOESM3_ESM.pdf]
